# Supplementary material for: Single-cell exon deletion profiling reveals splicing events that shape gene expression and cell state dynamics
Source: Nat Commun. 2026 Feb 3;17:1218. doi: 10.1038/s41467-026-68774-w (PMC12868714; doi:10.1038/s41467-026-68774-w)
Supplement: Supplementary file 1 — Supplementary Information [file 41467_2026_68774_MOESM1_ESM.pdf]

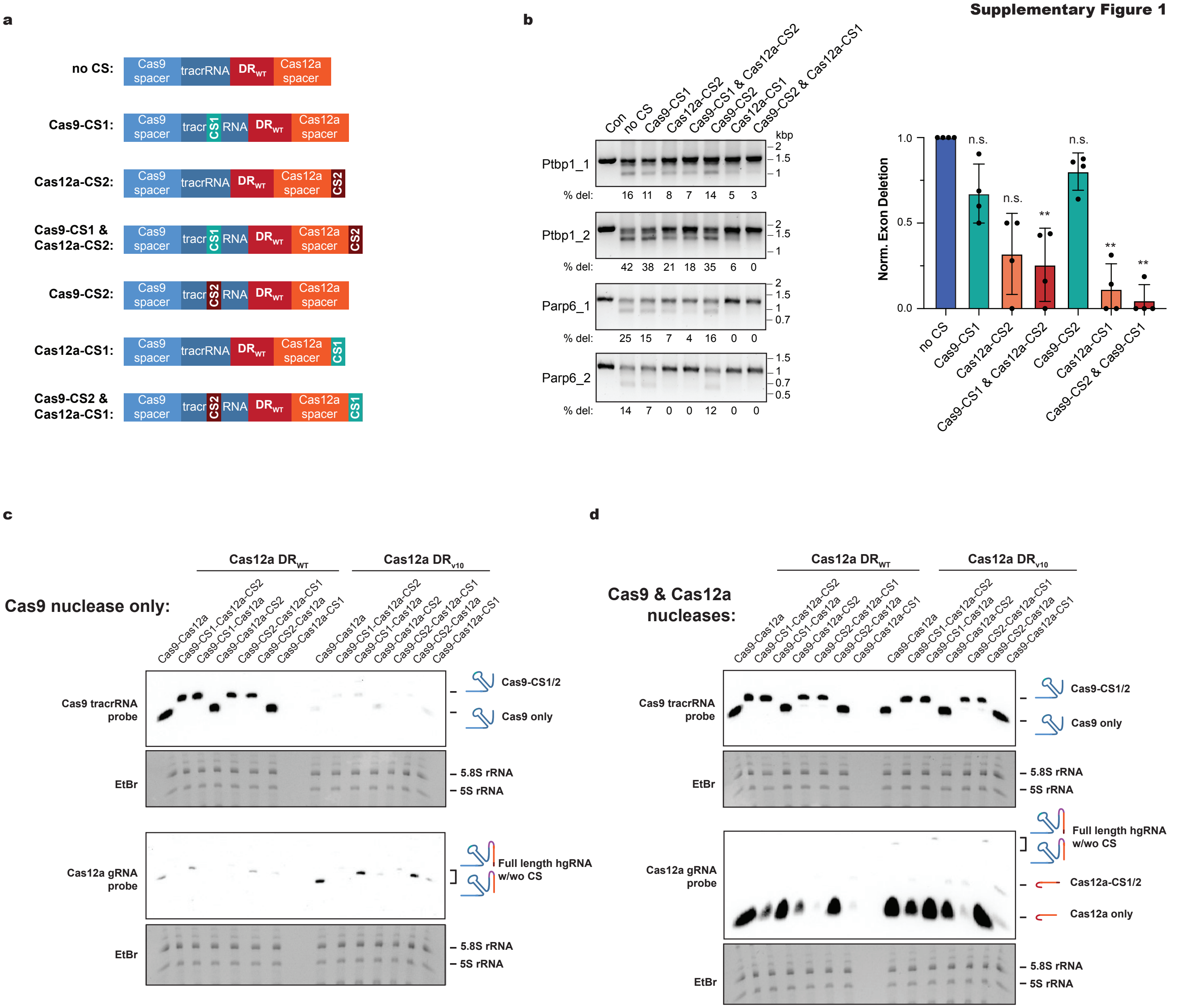

Supplementary Figure 1. Incorporation of capture sequences (CS) into the hgRNA reduces CHyMErA-mediated exon deletion efficiency and hgRNA expression.

(a) hgRNA guide modifications for testing gRNA expression and CHyMErA activity.

(b) CGR8 cells were transduced with the indicated hgRNA sequences, and deletion of *Ptbp1* exon-8 and *Parp6* exon-20 was evaluated on the DNA-level using PCR assays. Quantification of normalized exon deletion across four hgRNAs tested are displayed. Data represent mean ± SEM from four biological replicates. ** $p < 0.01$, * $p < 0.05$; one-way ANOVA.

(c, d) Northern blot analysis of Cas9 and Cas12a gRNAs generated from various hgRNA designs in HAP1 cells expressing only Cas9 (c) or both Cas9 and Cas12a nucleases (d). Upper panel uses a probe recognizing the tracrRNA and shows fully processed Cas9 gRNAs. Lower panel uses a probe recognizing the Cas12a spacer detecting both unprocessed hgRNAs and fully processed Cas12a gRNAs. Ethidium bromide (EtBr) staining was used as loading control.

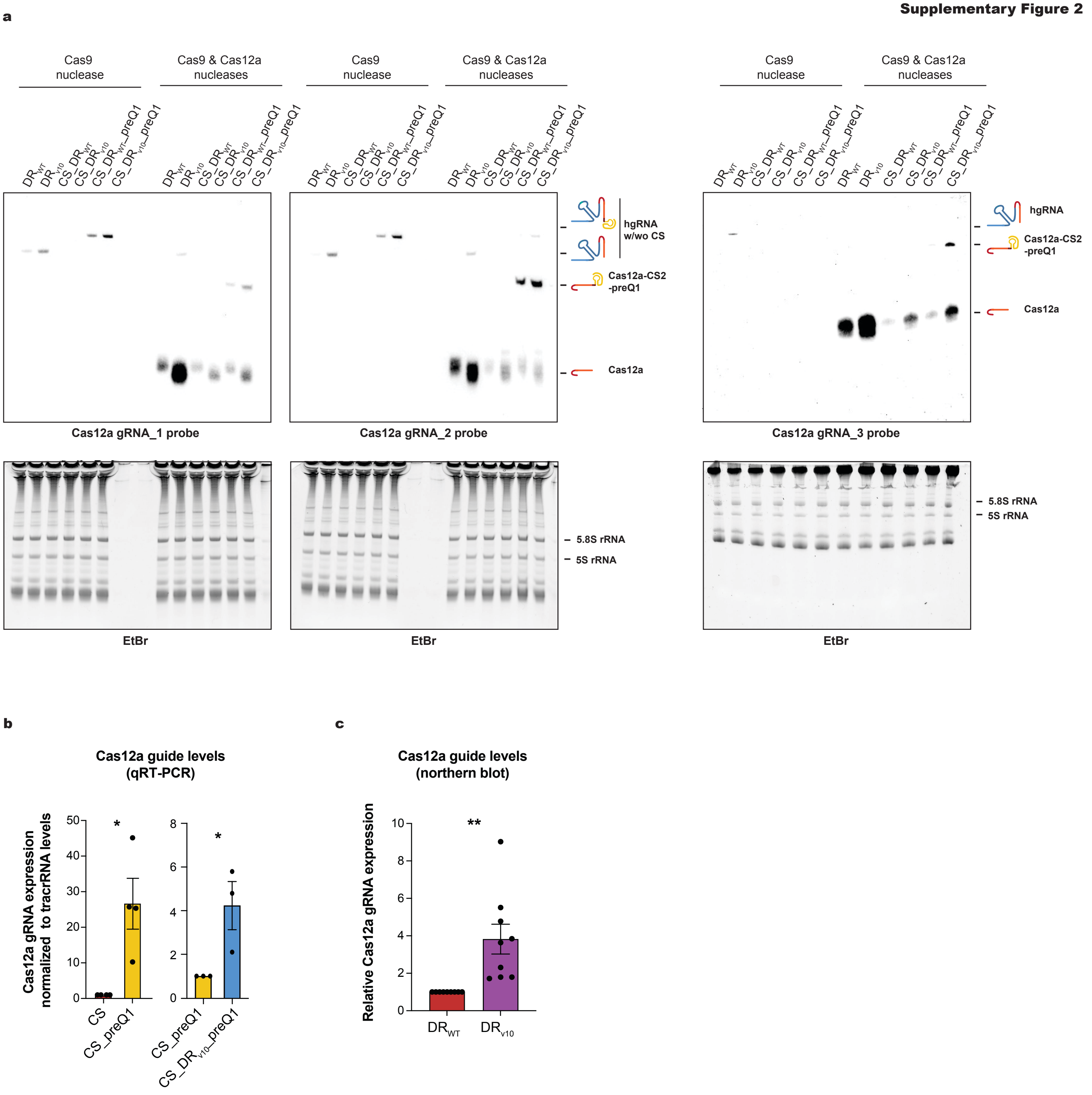

Supplementary Figure 2. The tevopreQ1 riboswitch and DR_{v10} enhance Cas12a gRNA expression.
(a) Northern blot analysis of total RNA extracted from HAP1 cells transduced with the indicated hgRNA constructs. A probe complementary to the Cas12a spacer sequence was used to assess gRNA expression levels (upper panel). Ethidium bromide (EtBr) staining was used as loading control (lower panel). Three distinct hgRNA sequences were tested, with each experiment performed in biological triplicate.
(b) Quantitative RT-PCR analysis of Cas12a guide RNA expression under different experimental conditions. In the left panel, primers targeting the Cas12a direct repeat (DR) and capture sequence 2 (CS2) were used. In the right panel, Cas12a guide-specific forward primers and a reverse primer recognizing the tevopreQ1 riboswitch were employed. Expression levels were normalized to endogenous Cas9 tracrRNA expression. Data represent mean ± SEM from biological replicates (n = 3-4). ** $p < 0.01$, * $p < 0.05$; two-tailed t-test.
(c) Quantification of processed Cas12a guide RNA expression comparing wild-type direct repeat (DR_{WT}) and DR_{v10} variants, based on the northern blot data shown in panel (a). Data represent mean ± SEM from n = 9 biological replicates. ** $p < 0.01$; two-tailed Wilcoxon test.

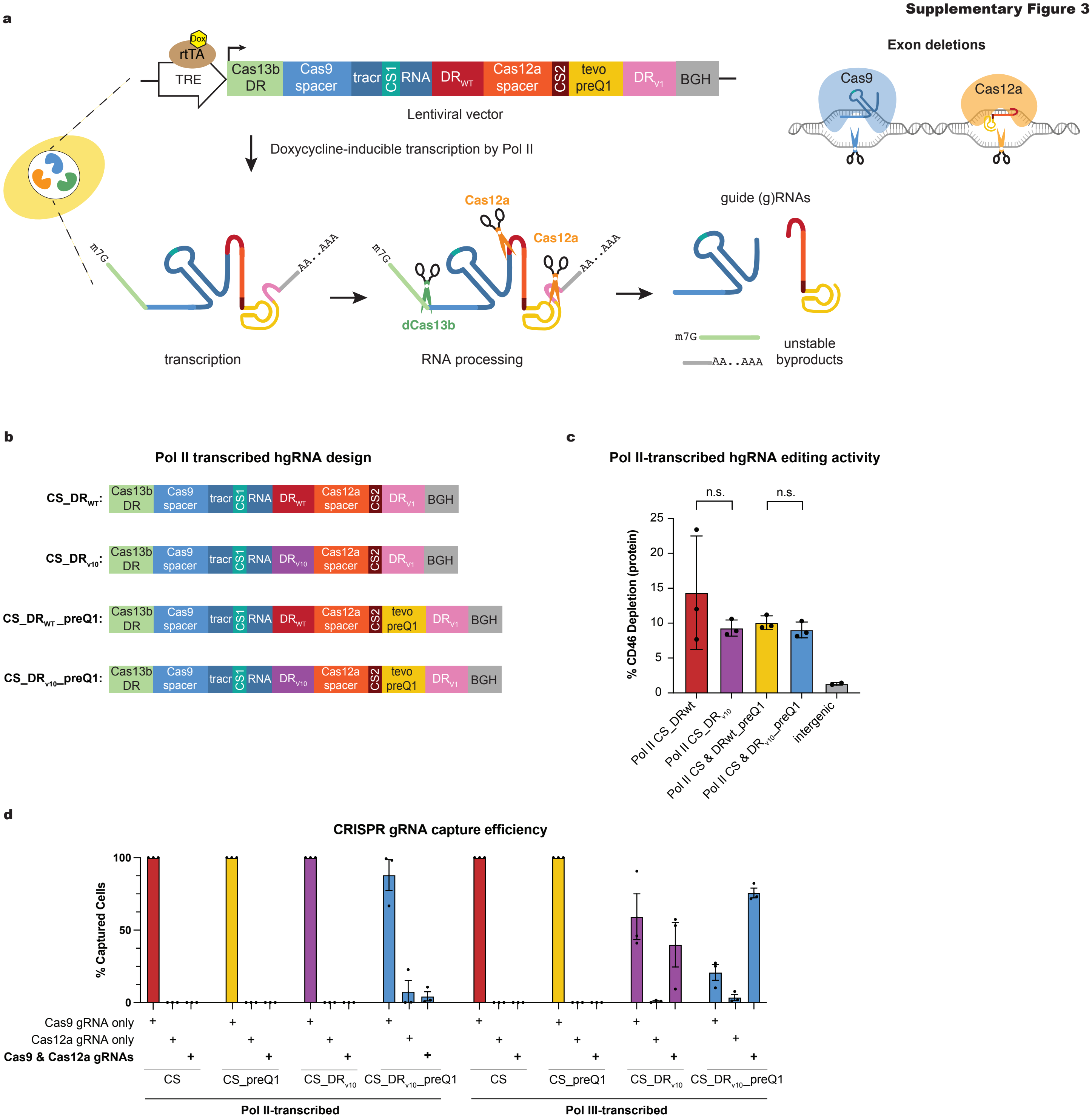

Supplementary Figure 3. DR_{v10} does not affect the activity of hgRNAs expressed from an RNA polymerase II promoter.
(a–b) Schematic of the system developed to express hgRNAs under a doxycycline-inducible RNA Pol II promoter (rTA-TRE). A Cas13b direct repeat (DR) was positioned at the 5′ end of the Cas9 gRNA to mediate precise processing and reconstitution of the hgRNA. Transcript polyadenylation was prevented by incorporating a Cas12a DR_{v1} sequence[35] at the 3′ end, minimizing recombination concerns. The hgRNA designs shown in (b) with three independent hgRNAs per condition, were transduced into HAP1 cells stably expressing Cas9, Cas12a, and d*Psp*Cas13d nucleases. Graphical elements were created in BioRender. Aregger, M. (2026) https://biorender.com/kloxl3s.
(c) Deletion of *CD46* exon 3 measured by flow cytometry in HAP1 cells transduced with three independent spacer pairs, comparing the indicated hgRNA designs. Data represent mean ± SEM from three biological replicates; significance was determined using a two-tailed t-test.
(d) Bar plots showing the proportions of cells in which Cas9 and/or Cas12a guide gRNAs were successfully detected using 10x Genomics droplet-based single-cell profiling. Different hgRNA designs, as indicated in Supplemental Figure 3b and Figure 1b, and Pol II- vs. Pol III-mediated expression were tested to assess their impact on gRNA capture efficiency. Data are normalized to the total number of cells with detectable gRNAs. Data represent mean ± SEM from three biological replicates.

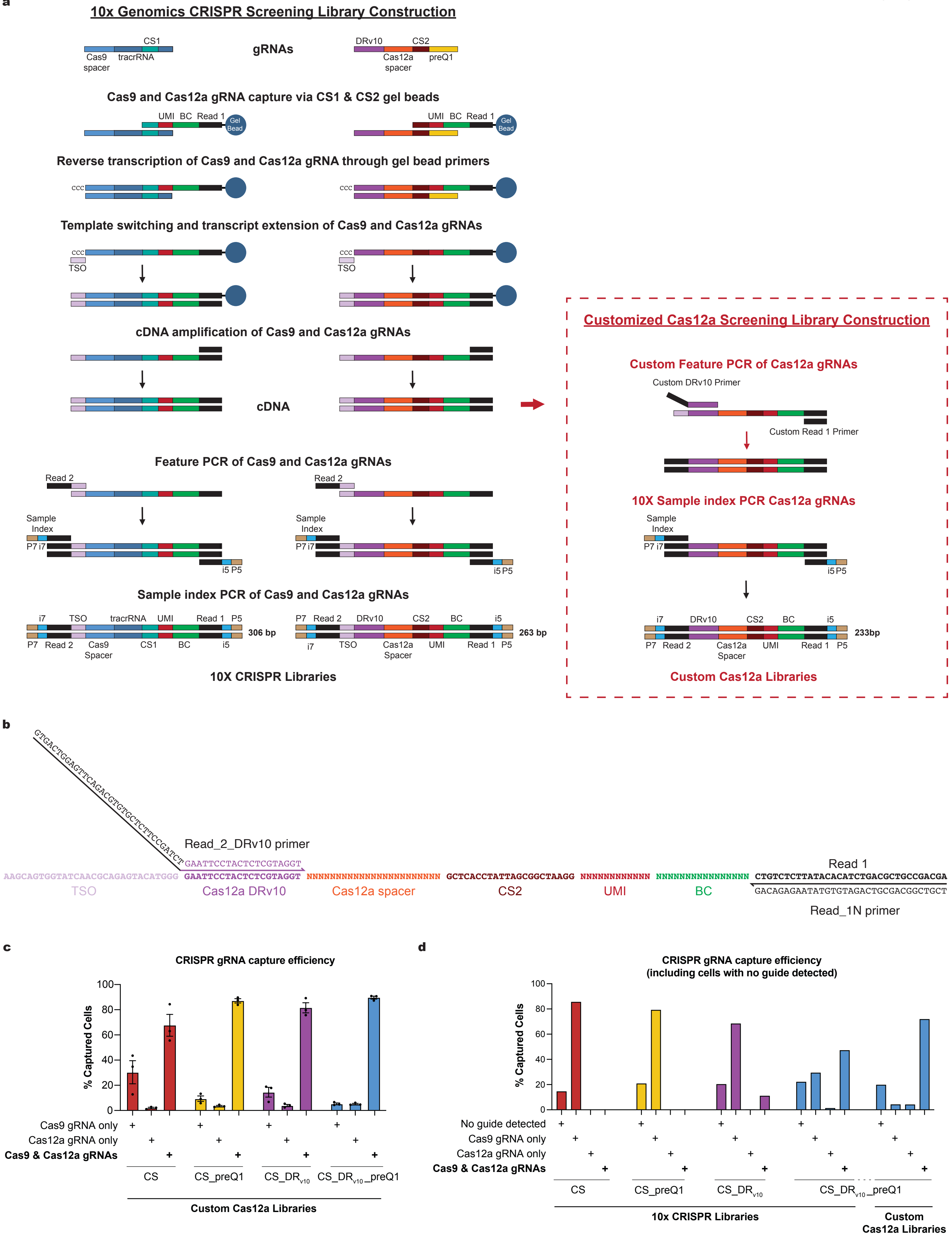
a
10x Genomics CRISPR Screening Library Construction
gRNAs
CS1
Cas9 spacer
tracrRNA
DRv10
CS2
Cas12a spacer
preQ1
Cas9 and Cas12a gRNA capture via CS1 & CS2 gel beads
UMI BC Read 1
Gel Bead
Reverse transcription of Cas9 and Cas12a gRNA through gel bead primers
ccc
Template switching and transcript extension of Cas9 and Cas12a gRNAs
TSO
cDNA amplification of Cas9 and Cas12a gRNAs
cDNA
Feature PCR of Cas9 and Cas12a gRNAs
Read 2
Sample Index
P7 i7
i5 P5
Sample index PCR of Cas9 and Cas12a gRNAs
i7 TSO tracrRNA UMI Read 1 P5
P7 Read 2 Cas9 Spacer CS1 BC i5
306 bp
P7 Read 2 DRv10 CS2 BC i5
i7 TSO Cas12a Spacer UMI Read 1 P5
263 bp
10X CRISPR Libraries
Customized Cas12a Screening Library Construction
Custom Feature PCR of Cas12a gRNAs
Custom DRv10 Primer
Custom Read 1 Primer
10X Sample index PCR Cas12a gRNAs
Sample Index
P7 i7
i5 P5
i7 DRv10 CS2 BC i5
P7 Read 2 Cas12a Spacer UMI Read 1 P5
233bp
Custom Cas12a Libraries
b
GTGACTGGAGTTCAGACGTGTGCTCTTCCGATCT
Read_2_DRv10 primer
GAATTCCTACTCTCGTAGGT
AAGCAGTGGTATCAACGCAGAGTACATGGG GAATTCCTACTCTCGTAGGT NNNNNNNNNNNNNNNNNNNNNN GCTCACCTATTAGCGGCTAAGG NNNNNNNNNNNN NNNNNNNNNNNNNNNN CTGTCTCTTATACACATCTGACGCTGCCGACGA
TSO
Cas12a DRv10
Cas12a spacer
CS2
UMI
BC
Read 1
GACAGAGAATATGTGTAGACTGCGACGGCTGCT
Read_1N primer
c
CRISPR gRNA capture efficiency
% Captured Cells
0
20
40
60
80
100
Cas9 gRNA only
Cas12a gRNA only
Cas9 & Cas12a gRNAs
CS
CS_preQ1
CS_DRv10
CS_DRv10_preQ1
Custom Cas12a Libraries
d
CRISPR gRNA capture efficiency
(including cells with no guide detected)
% Captured Cells
0
20
40
60
80
100
No guide detected
Cas9 gRNA only
Cas12a gRNA only
Cas9 & Cas12a gRNAs
CS
CS_preQ1
CS_DRv10
CS_DRv10_preQ1
10x CRISPR Libraries
Custom Cas12a Libraries

Supplementary Figure 4. Schematic representation of Cas9 and Cas12a gRNA library preparation for scCHyMErA-Seq.
(a) Workflow for generating Cas9 and Cas12a gRNA libraries for Illumina sequencing in the scCHyMErA-Seq framework. The left panel shows the standard 10x Genomics CRISPR Screening library construction, which efficiently captures Cas9 but not Cas12a gRNAs. To enable simultaneous detection of both, scCHyMErA-Seq incorporates an additional Cas12a-specific enrichment step (right panel). A custom primer (Read2_DRv10) selectively enriches Cas12a gRNAs from 10x Genomics CRISPR cDNA. Following feature amplification, samples are indexed using standard 10x Genomics reagents, and custom Cas12a libraries are sequenced alongside standard 10x Genomics CRISPR libraries. CS, capture sequence; DR_{v10}, Cas12a direct repeat variant 10; preQ1, tevopreQ1 riboswitch; UMI, unique molecular identifier; BC, 10x Genomics cell barcode; TSO, template switch oligo; Read 1+2, Illumina primer annealing sites; i5+i7, Illumina sample barcodes; P5+P7, Illumina flow-cell adaptors.
(b) Schematic depicting the sequences of the custom primers used for specific amplification of Cas12a gRNAs from CRISPR cDNA. The Read_2_DRv10 primer binds to the Cas12a DRv10 sequence and includes a Read 2 overhang, while the Read_1N primer anneals to the Read 1 sequence.
(c) Bar plots showing the proportions of cells in which Cas9 and/or Cas12a guide gRNAs were successfully detected using droplet-based single-cell profiling with the final scCHyMErA-Seq protocol, which includes custom Cas12a gRNA library generation (as described in (a)). Different hgRNA designs, as indicated in Figure 1b, were tested to assess their impact on gRNA capture efficiency. Data are normalized to the total number of cells with detectable gRNAs. Data represent mean ± SEM from biological replicates.
(d) Bar plots showing the overall proportions of cells in which Cas9 and/or Cas12a guide gRNAs were successfully detected across all profiled cells using 10x Genomics droplet-based single-cell transcriptomics. The same data is shown as in Fig. 1d, but here the data is normalized to the total of all profiled cells, including those where no gRNAs were captured. See also Supplementary Data 1.

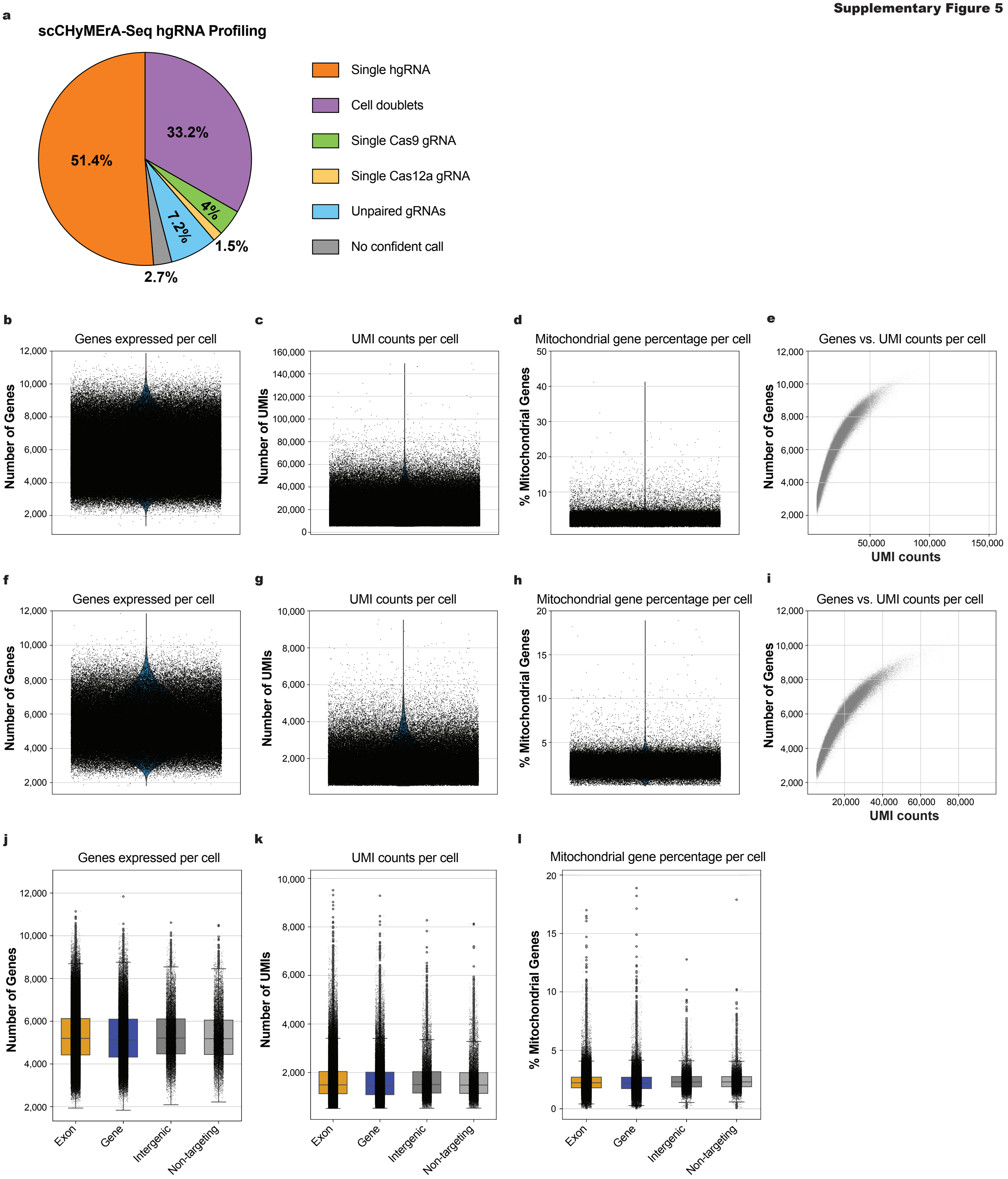
a
scCHyMErA-Seq hgRNA Profiling
51.4%
33.2%
4%
7.2%
1.5%
2.7%
Single hgRNA
Cell doublets
Single Cas9 gRNA
Single Cas12a gRNA
Unpaired gRNAs
No confident call
b
Genes expressed per cell
Number of Genes
c
UMI counts per cell
Number of UMIs
d
Mitochondrial gene percentage per cell
% Mitochondrial Genes
e
Genes vs. UMI counts per cell
Number of Genes
UMI counts
f
Genes expressed per cell
Number of Genes
g
UMI counts per cell
Number of UMIs
h
Mitochondrial gene percentage per cell
% Mitochondrial Genes
i
Genes vs. UMI counts per cell
Number of Genes
UMI counts
j
Genes expressed per cell
Number of Genes
Exon
Gene
Intergenic
Non-targeting
k
UMI counts per cell
Number of UMIs
Exon
Gene
Intergenic
Non-targeting
l
Mitochondrial gene percentage per cell
% Mitochondrial Genes
Exon
Gene
Intergenic
Non-targeting

Supplementary Figure 5. Quality control analysis of scCHyMErA-Seq data.
(a) Pie chart showing the distribution of cell droplets expressing either a single hgRNA or multiple hgRNAs. Only cells expressing a single hgRNA were retained for downstream analysis.
(b–i) Box plots summarizing key quality control metrics before (b–e) and after (f–i) filtering for cells expressing a single hgRNA. Metrics include the number of unique genes detected per cell (b, f), total unique molecular identifier (UMI) counts per cell (c, g), percentage of mitochondrial gene expression (d, h), and the correlation of UMI counts for each expressed gene in each cell (e, i).
(j-l) Box plots summarizing key quality control metrics across cells expressing different categories of hgRNAs. Metrics include the number of unique genes detected per cell (j), total unique molecular identifier (UMI) counts per cell (k), and the percentage of mitochondrial gene expression (l). Boxes show interquartile range (IQR), 25th to 75th percentile, with the median indicated by a horizontal line. Whiskers extend to the quartile ± 1.5 × IQR.

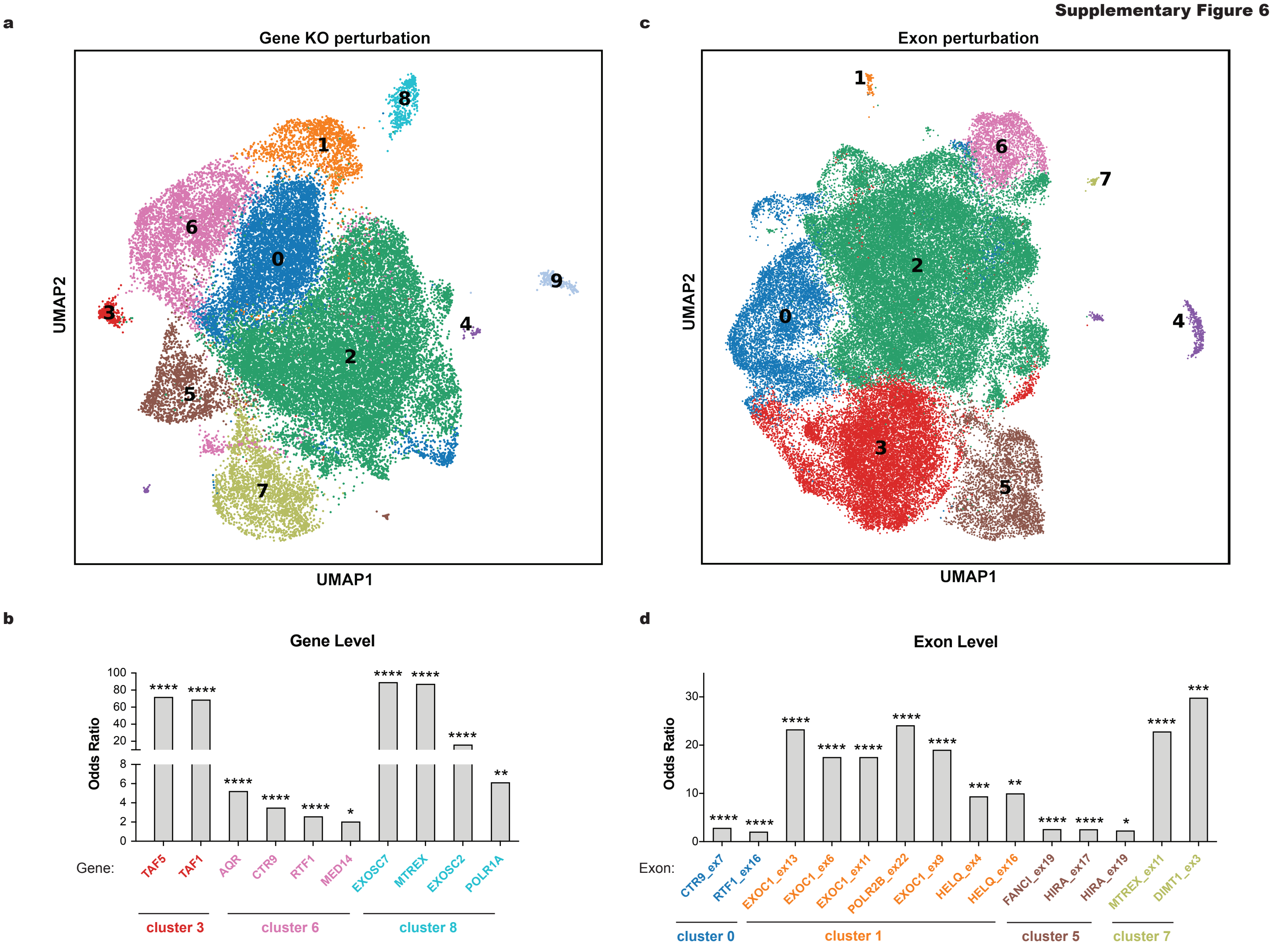

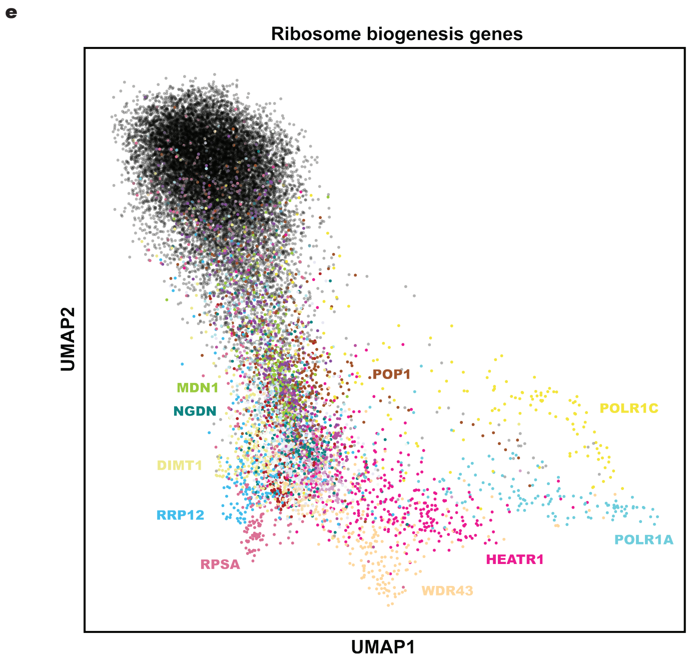

Supplementary Figure 6. Reduced dimensionality visualization of scCHyMErA-Seq data.

(a, c) UMAP plots showing the spatial distribution of cells following gene-level (a) and exon-level (c) perturbations, clustered using Leiden analysis.

(b, d) Enrichment analysis of gRNAs targeting specific genes (b) or exons (d) within individual Leiden clusters shown in (a) and (c). Enrichment was calculated using two-sided Fisher's exact test with Holm correction. Significance is indicated as: **** $p < 0.0001$, *** $p < 0.001$, ** $p < 0.01$, *$p < 0.05$.

(e) UMAP visualization of cells expressing hgRNAs targeting ribosome biogenesis factors, following dimensionality reduction with linear discriminant analysis (LDA).

a

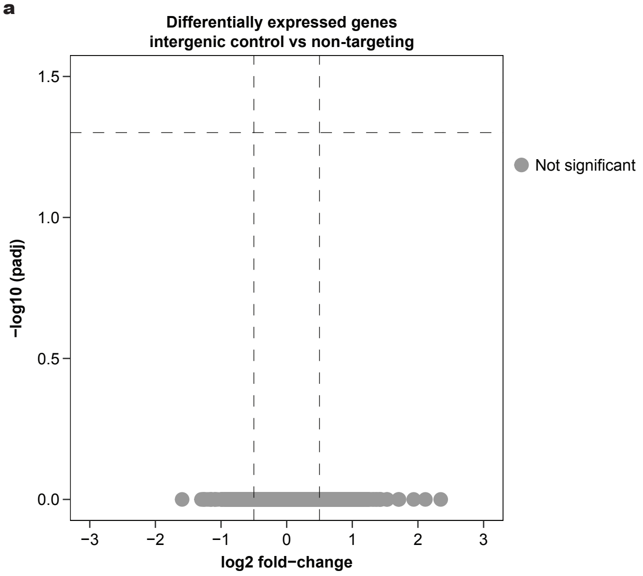

b

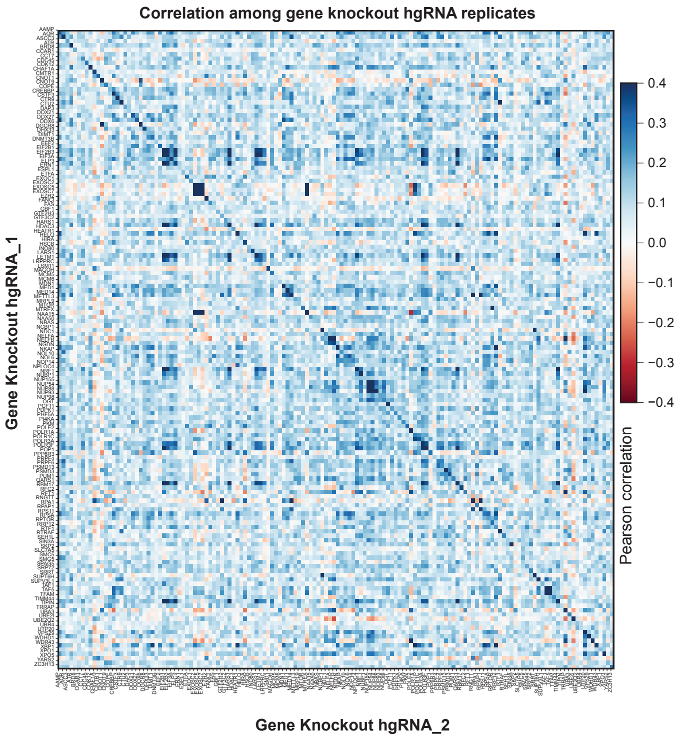

c

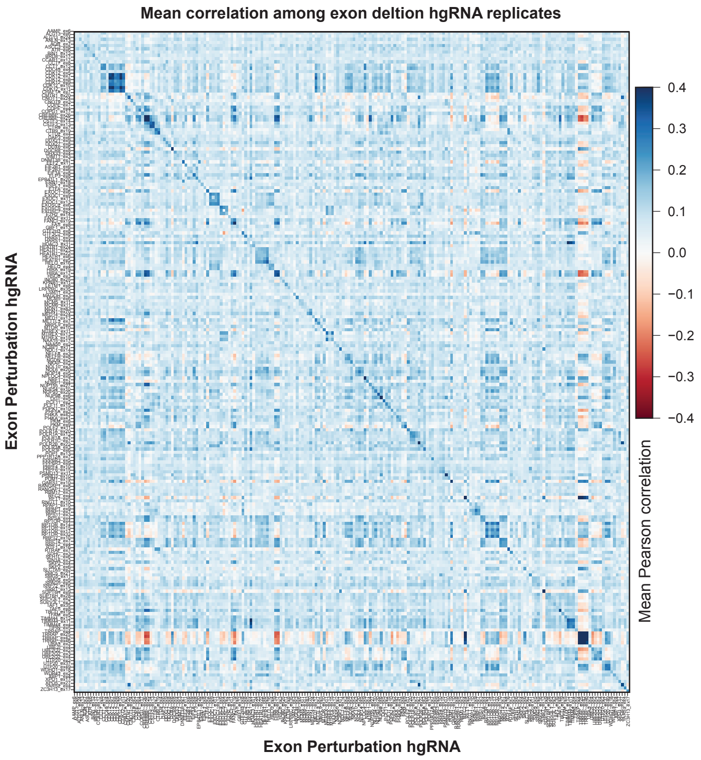

Supplementary Figure 7. Differential gene expression analysis across control hgRNAs and replicates.
(a) Volcano plot showing DESeq2 aggregate differential expression results. Each point represents a gene, comparing cells carrying intergenic control versus non-targeting control hgRNAs. p-values were computed using DESeq2's Wald test with Benjamini–Hochberg correction.
(b, c) Heatmaps illustrating replicate correlations for gene knockout (b) and exon deletion hgRNAs (c). Color intensity reflects Pearson correlation values for differentially expressed genes between the different hgRNA sequences.

Supplementary Figure 8

Differential gene expression plots from gene-level knockout perturbations

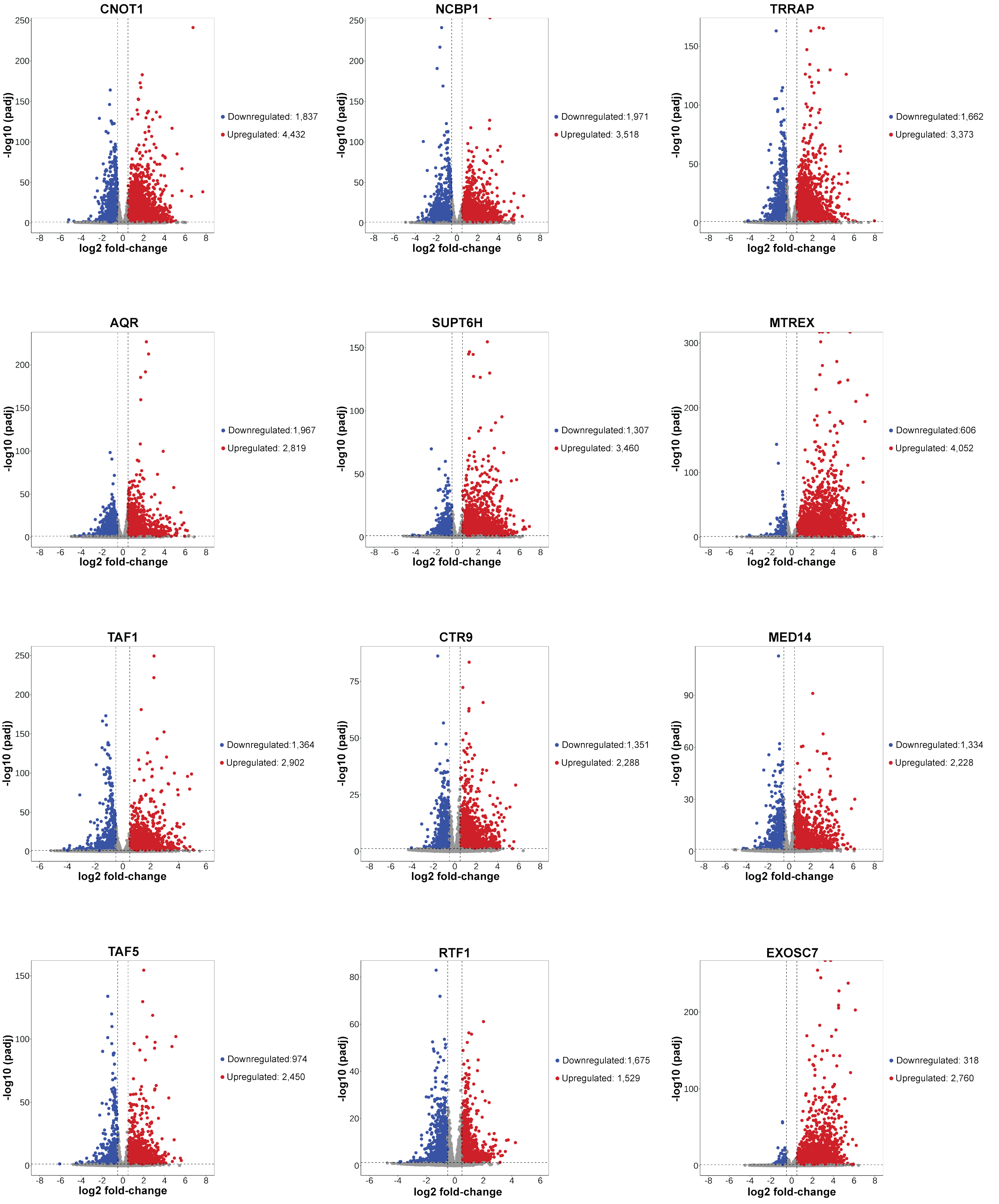

Supplementary Figure 8. Differential gene expression following gene-level knockout perturbations.
Volcano plot showing DESeq2 pseudobulk differential expression analysis comparing cells carrying the indicated gene knockouts with cells transduced with non-targeting intergenic control hgRNAs. Each point represents a gene; significant upregulated genes are shown in red and downregulated genes in blue (adjusted p-value < 0.05 and |log2 fold-change| > 0.5). p-values were computed using DESeq2's Wald test with Benjamini–Hochberg correction. Full differential expression results are provided in Supplementary Data 3.

Supplementary Figure 9

Differential gene expression plots from representative exon deletion perturbations

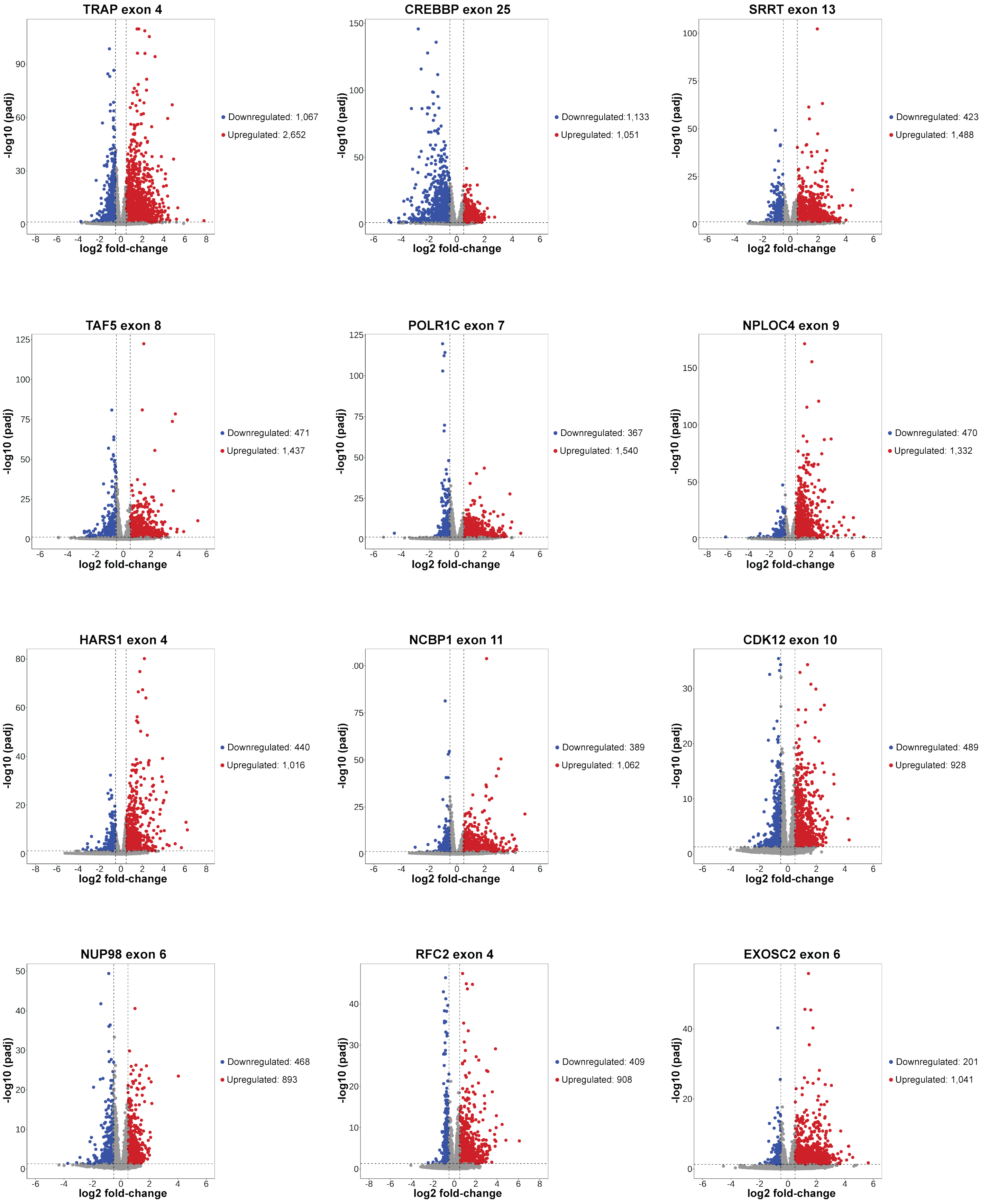

Supplementary Figure 9. Differential gene expression following exon deletion perturbations.
Volcano plot showing DESeq2 pseudobulk differential expression analysis comparing cells carrying the indicated exon deletions with cells transduced with non-targeting intergenic control hgRNAs. Each point represents a gene; significant upregulated genes are shown in red and downregulated genes in blue (adjusted p-value < 0.05 and |log2 fold-change| > 0.5). p-values were computed using DESeq2's Wald test with Benjamini–Hochberg correction. Complete differential expression results are provided in Supplementary Data 4.

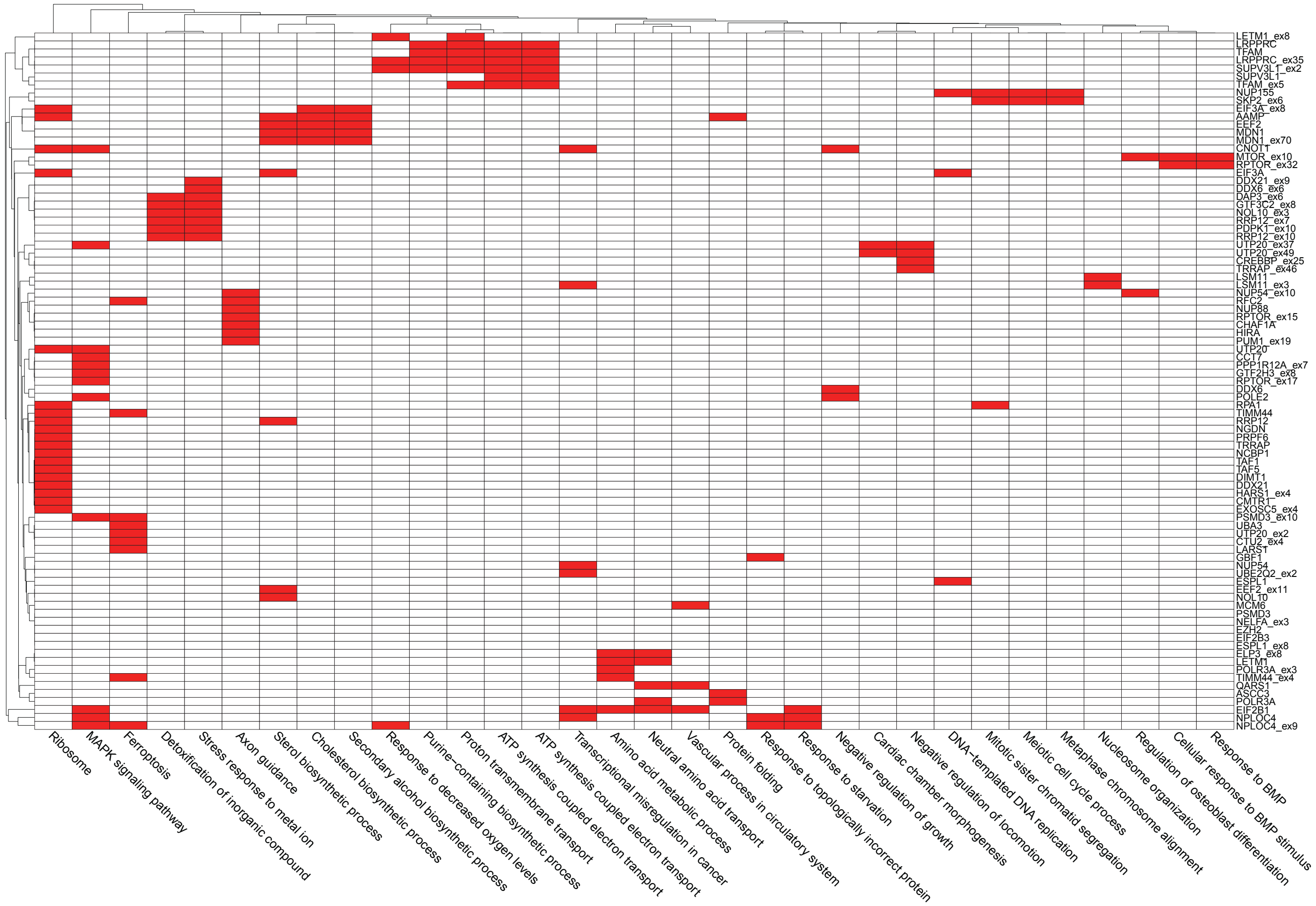

Supplementary Figure 10. Gene ontology–based functional enrichment.
Gene Ontology (GO) enrichment analysis of genes identified as differentially expressed by the scCHyMErA-Seq screen following the indicated perturbations. The visualization highlights significantly enriched biological processes associated with specific gene knockout and exon deletion (_ex) perturbations (p_{adj} < 0.05, Fisher's exact one-tailed test with multiple-testing corr).

Supplementary Figure 11

a

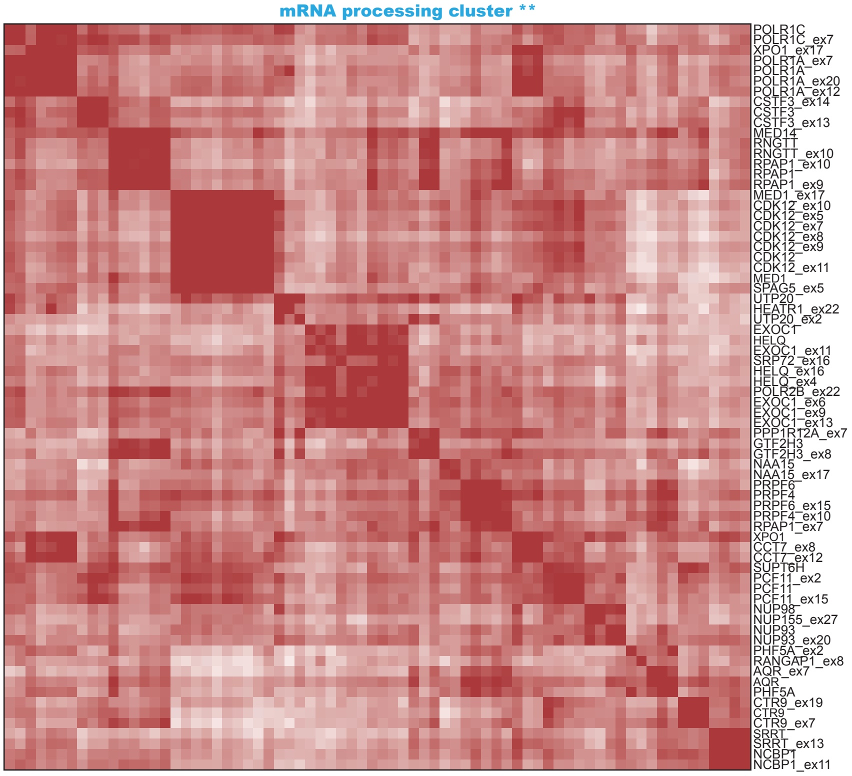

b

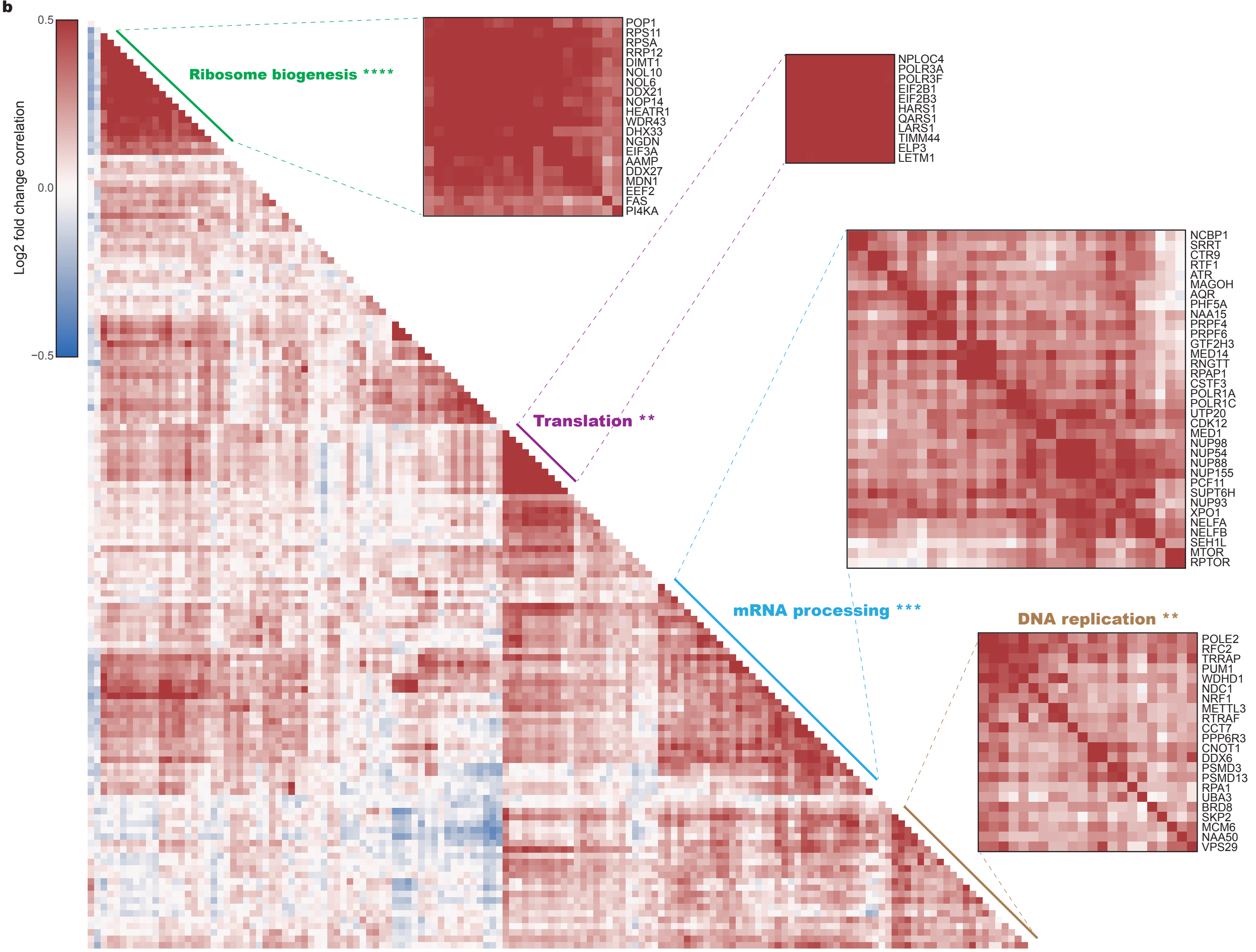

Supplementary Figure 11. Clustering of gene functions from scCHyMErA-Seq transcriptional profiling.
(a) Subcluster from Figure 3b highlighting genes enriched for mRNA processing pathways, annotated using the Molecular Signatures Database (MSigDB); enrichment significance is indicated ($p < 0.01$). Perturbed genes and exons (_ex) are listed on the right.
(b) Heatmap showing pairwise correlations of log2 fold-change values for differentially expressed genes across gene-level knockout perturbations identified by scCHyMErA-Seq. Distinct subclusters are annotated with MSigDB pathway terms: **** $p < 0.0001$, *** $p < 0.001$, ** $p < 0.01$; one-sided Fisher's exact test corrected for multiple hypothesis (Benjamini-Hochberg adjusted). Representative genes from each subcluster are listed on the right.

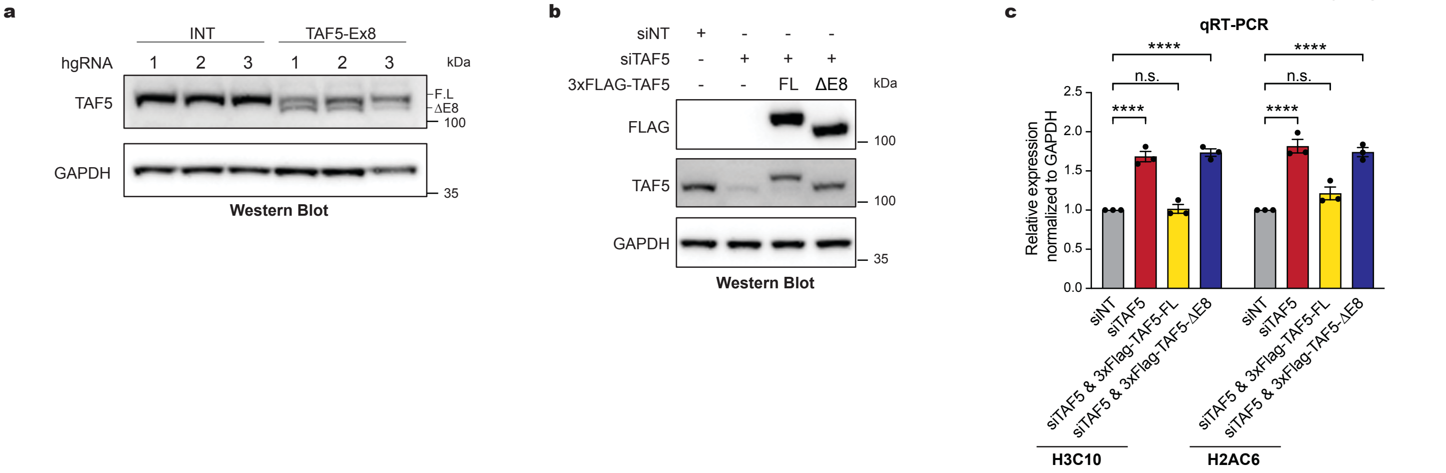

Supplementary Figure 12. Deletion of TAF5 exon-8 and LSM11 exon-3 leads to correlated gene expression responses.

(a) Western blot analysis of HAP1 cells transduced with three independent intergenic control hgRNAs or three independent hgRNAs targeting *TAF5* exon-8. Exon deletions were generated using constructs compatible with the scCHyMErA-Seq system. The two TAF5 protein isoforms (full-length, FL; and exon-8-deleted, ΔE8) are indicated. GAPDH served as a loading control. Representative images from two independent experiments.

(b) Western blot analysis of TAF5 isoform expression in HEK293 Flp-In cell lines stably expressing doxycycline-inducible 3×FLAG-tagged TAF5 isoforms with exon-8 included (FL) or excluded (ΔE7). The ectopically expressed isoforms contain silent mutations introduced at the siRNA target site, rendering them resistant to siNRF1 treatment. Cells were transfected with either control siRNAs (siNT) or siRNAs targeting endogenous TAF5 (siTAF5). Blots were probed with antibodies against TAF5, FLAG, and GAPDH (loading control). Representative images from two independent experiments.

(c) qRT-PCR analysis of selected replication-dependent histone genes in HEK293 Flp-In cell lines stably expressing doxycycline-inducible, siRNA-resistant 3×FLAG-tagged TAF5 cDNAs with exon-8 either included (FL) or excluded (ΔE8). Cells were treated with control siRNAs (siNT) or siRNA targeting endogenous TAF5 (siTAF5). Data are presented as mean relative expression normalized to GAPDH ±

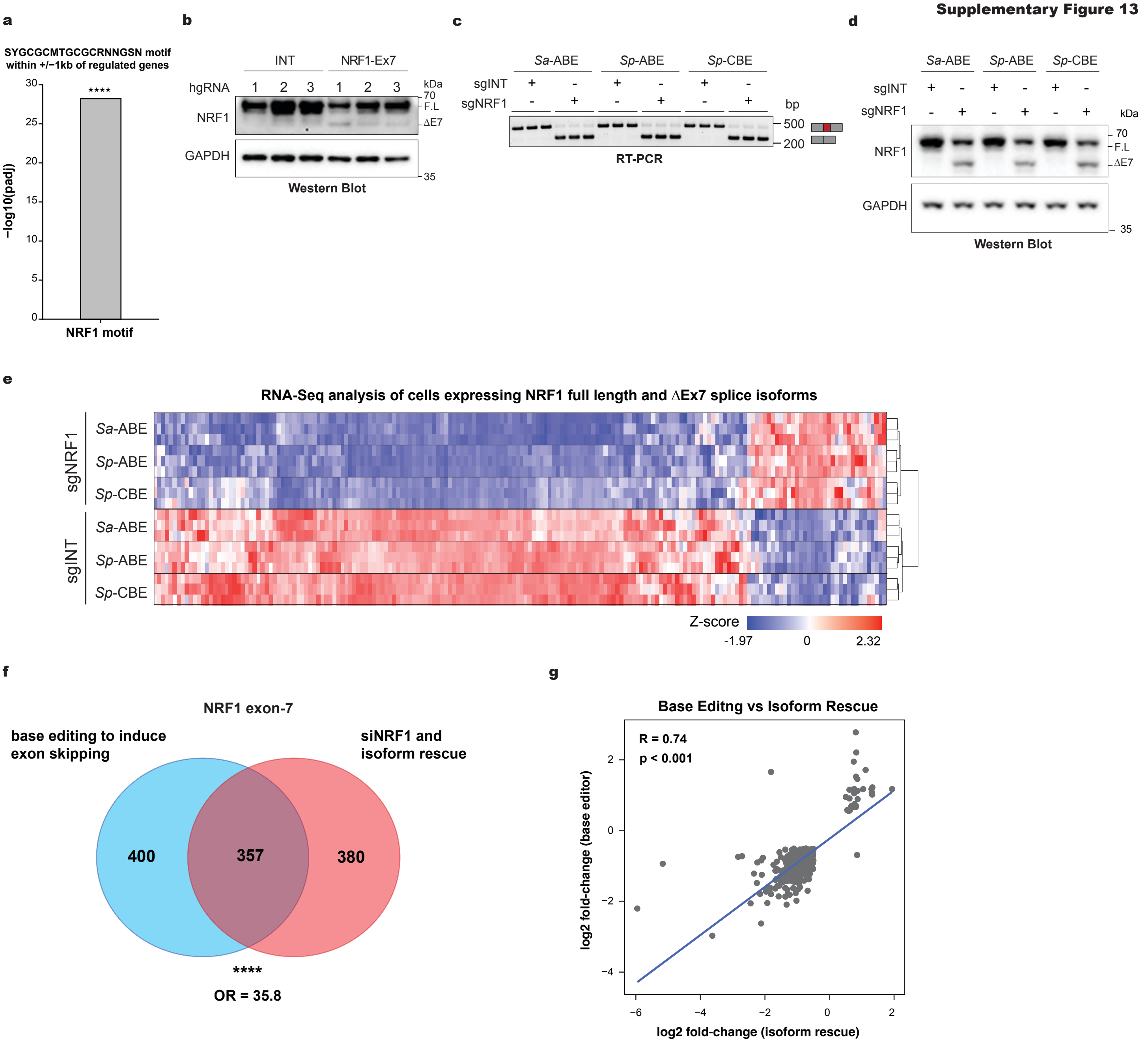

Supplementary Figure 13. Deletion of NRF1 alternative exon 7 induces significant changes in gene expression.
(a) Motif enrichment analysis of differentially expressed genes reveals significant enrichment of the NRF1 consensus binding motif ($p < 0.0001$, hypergeometric testing). All expressed genes are used as background.
(b) Western blot analysis of HAP1 cells transduced with three independent intergenic control hgRNAs or three independent hgRNAs targeting *NRF1* exon-7. Exon deletions were generated using constructs compatible with the scCHyMErA-Seq system. The two TAF5 protein isoforms (full-length, FL; and exon-7-deleted, ΔE7) are indicated. GAPDH served as a loading control.
(c) RT-PCR analysis of HEK293T cells transfected with plasmids encoding base editors and Cas9 gRNAs targeting the *NRF1* exon-7 splice site (sgNRF1), inducing exon skipping. Cells transfected with an intergenic control gRNA (sgINT) served as controls. *Sa: S. aureus Cas9; Sp: S. pyogenes* Cas9; ABE: adenine base editor; CBE: cytosine base editor.
(d) Western blot analysis of NRF1 protein levels in HEK293T cells transfected with base editor plasmids and either NRF1 exon-7 splice site-targeting gRNAs or intergenic control gRNAs. Blots were probed with antibodies specific for NRF1; GAPDH was used as a loading control. Single experiment performed.
(e) RNA-seq analysis showing Z-score normalized expression changes of differentially expressed genes in HEK293T cells transfected with base editor gRNAs inducing *NRF1* exon-7 skipping compared to intergenic controls.
(f) Venn diagram depicting the overlap of genes regulated by NRF1 exon-7, identified through bulk RNA-seq analysis of HEK293 Flp-In cells expressing either full-length or Δexon-7 NRF1 isoforms, and HEK293T cells engineered to express NRF1-Δex7 using base editing. Odds Ratio (OR) is indicated ($p < 0.0001$; Fisher's exact test).
(g) Two-sided pearson correlation plot comparing log2 fold-changes of differentially expressed genes following NRF1 exon-7 skipping in isoform-rescue experiments versus base editing experiments.

Supplementary Figure 14

a

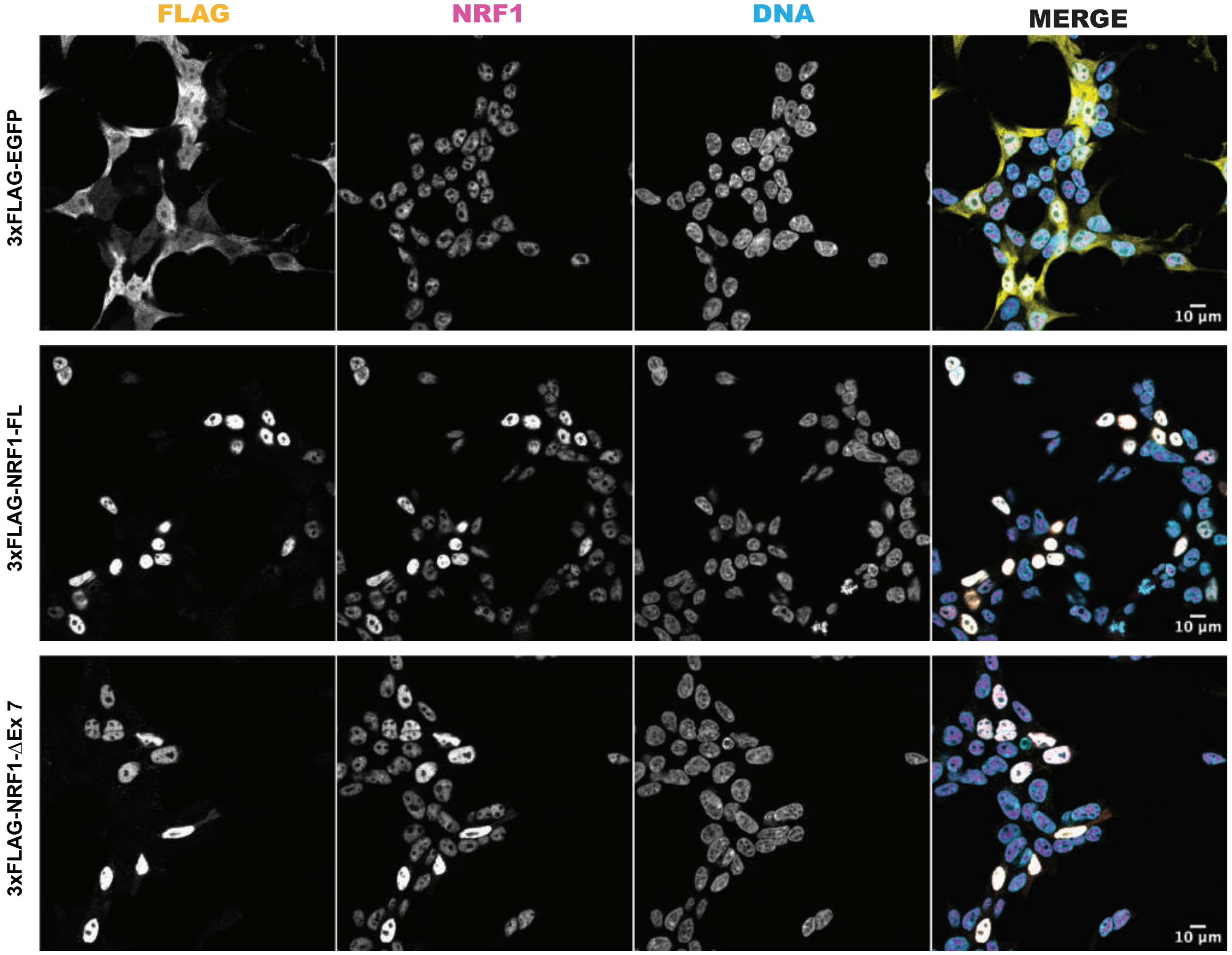

b

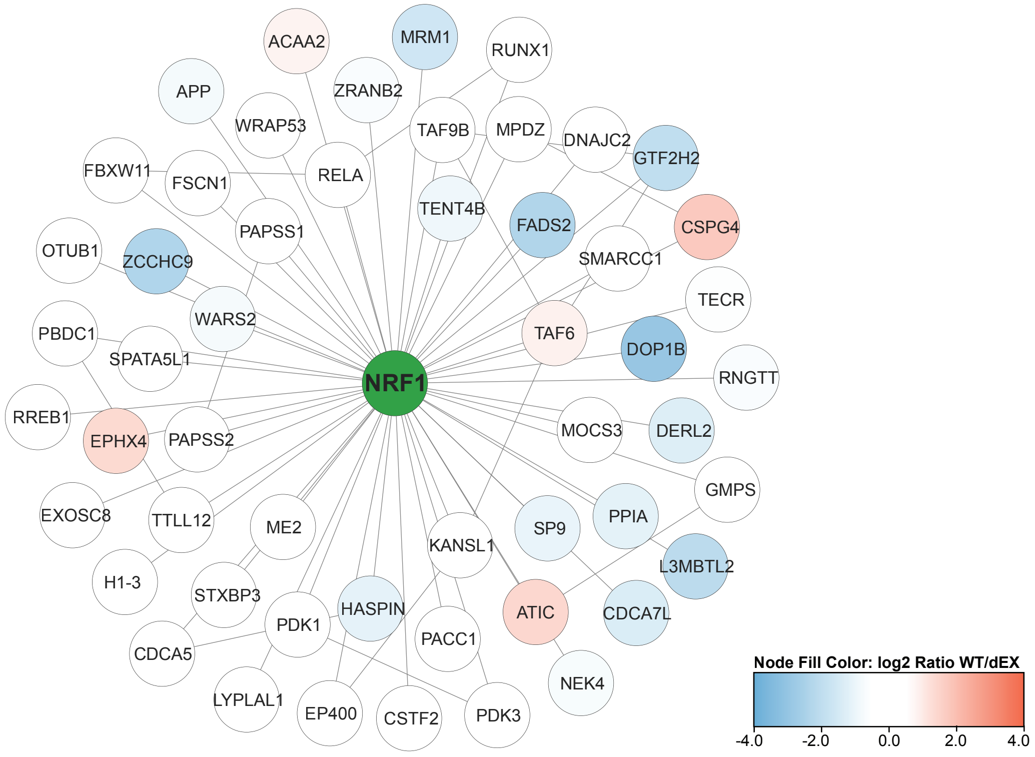

Supplementary Figure 14. NRF1 exon-7 has no impact on its subcellular localization or protein-protein interactions.
(a) Immunofluorescence imaging of NRF1 splice isoforms (FL and ΔEx7) in HEK293 Flp-In cells following 24 hours of doxycycline induction. NRF1 was detected using FLAG (yellow) or NRF1 (pink) antibodies, and nuclei were counterstained with Hoechst 33342 (cyan). Scale bar: 10 μm. Single experiment performed
(b) Protein-protein interaction network of NRF1 splice isoforms detected by miniTurboID-mass spectrometry. Node color indicates differential protein associations between the two isoforms. Edges represent protein-protein interactions retrieved from the STRING database, connecting preys identified by proximity labeling.

Supplementary Figure 15

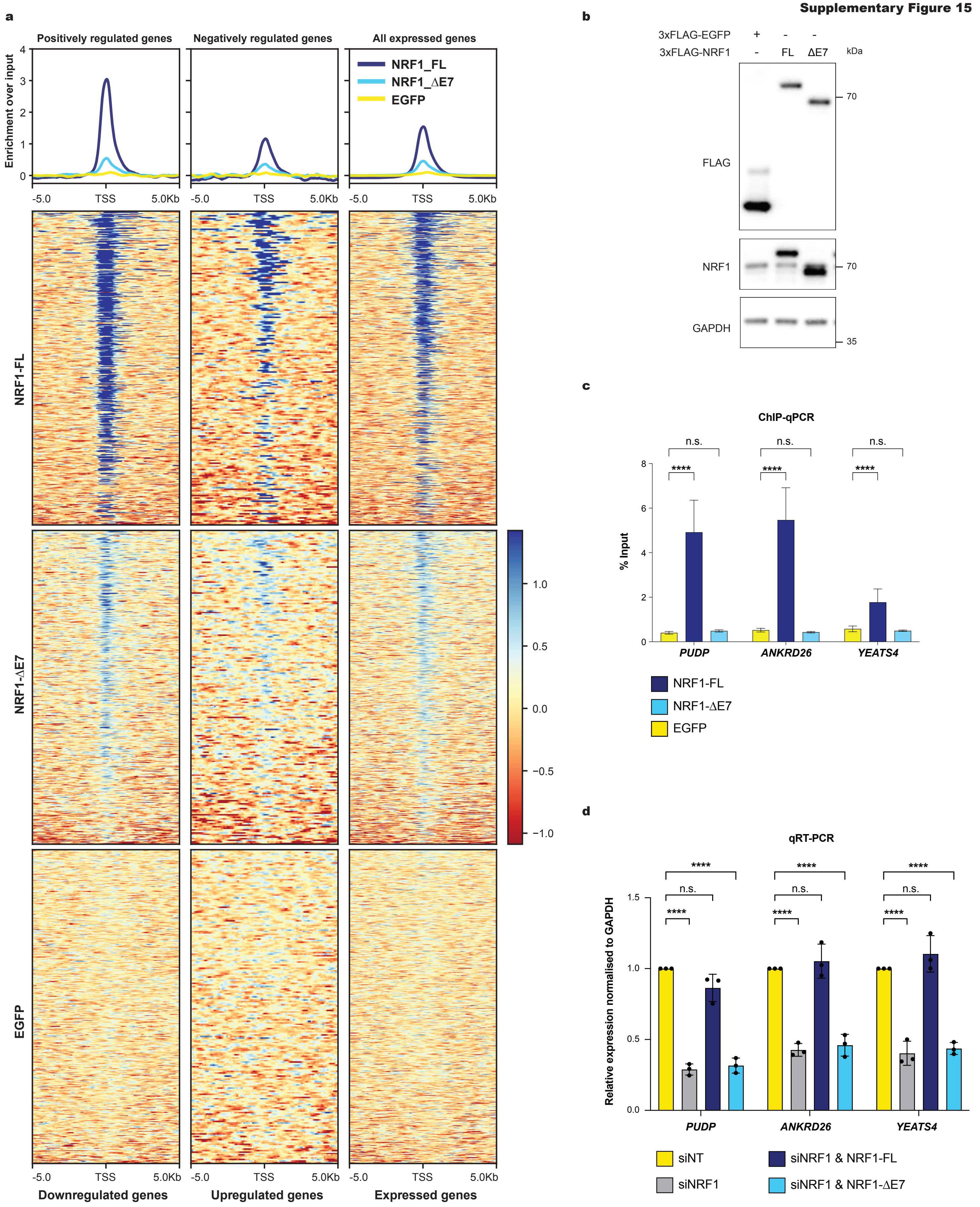

Supplementary Figure 15. NRF1 exon-7 modulates NRF1 DNA-binding activity.
(a) ChIP-seq analysis comparing NRF1 full-length (FL) and ∆exon 7 (∆E7) isoforms. Density plots (enrichment over input) and genomic heatmaps show ChIP peak intensities centered on transcriptional start sites (TSS) extending ±5 kb. Separate profiles are presented for genes upregulated, downregulated, or unaffected by NRF1 exon-7 skipping (see Figure 4b).
(b) Western blot analysis of NRF1 isoform expression in HEK293 Flp-In cell lines stably expressing doxycycline-inducible 3×FLAG-tagged NRF1 isoforms containing exon 7 (FL) or lacking exon 7 (ΔE7), used in the ChIP-qPCR assays. Blots were probed with antibodies against NRF1, FLAG, and GAPDH (loading control). Representative images from three independent experiments.
(c) ChIP-qPCR analysis of NRF1-FL and NRF1-∆E7 binding at promoter regions of the indicated target genes in HEK293 Flp-In cells. Data are shown as mean percent over input ± SEM of three biological replicates. Statistical comparisons between control (siNT) and experimental conditions are indicated; **** $p < 0.0001$ (two-way ANOVA).
(d) qRT-PCR analysis of selected NRF1 exon-7-regulated genes in HEK293 Flp-In cell lines. Cells were treated with either siNRF1 or a non-targeting control and stably expressed doxycycline-inducible, siRNA-resistant 3×FLAG-tagged NRF1 cDNAs, with exon-7 either included (FL) or excluded (∆E7). Data are presented as mean relative expression normalized to GAPDH ± SEM of three biological replicates. **** $p < 0.0001$ (two-way ANOVA).

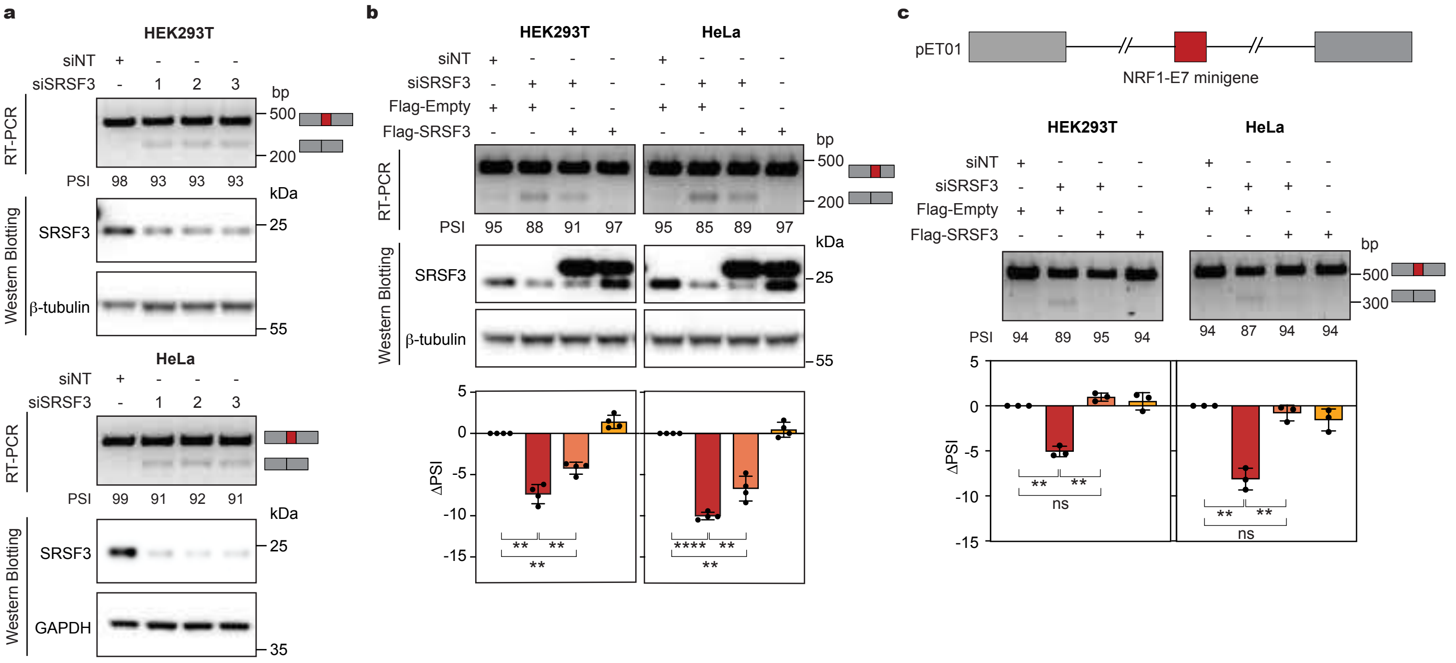

Supplementary Figure 16. SRSF3 promotes inclusion of NRF1 exon-7.

(a) RT-PCR analysis of endogenous NRF1 exon-7 splicing in HEK293 and HeLa cells transfected with three independent siRNAs or a pooled siRNA targeting SRSF3. Percent Spliced In (PSI) values are shown. Knockdown of SRSF3 was monitored by western blot analysis (lower panels). Blots were probed with antibodies specific for SRSF3; β-tubulin was used as a loading control. Single experiment performed.

(b) RT-PCR analysis of endogenous NRF1 exon-7 splicing in HEK293T and HeLa cells transfected with an siRNA targeting SRSF3 and/or a siRNA-resistant SRSF3 cDNA expression construct (upper panel). PSI values are indicated, and ΔPSI reflects the difference between control and experimental conditions across four independent experiments (lower panel). Knockdown and rescue of SRSF3 was monitored by western blot analysis (middle panels). Blots were probed with antibodies specific for SRSF3; β-tubulin was used as a loading control.

(c) RT-PCR analysis of NRF1 exon-7 splicing using minigene reporters transfected into HEK293T cells following SRSF3 knockdown and/or rescue with siRNA-resistant SRSF3 cDNA. ΔPSI values are displayed for three biological replicates.

Data are presented as mean ± SD; **** $p < 0.0001$, ** $p < 0.01$ (two-tailed paired t-test).

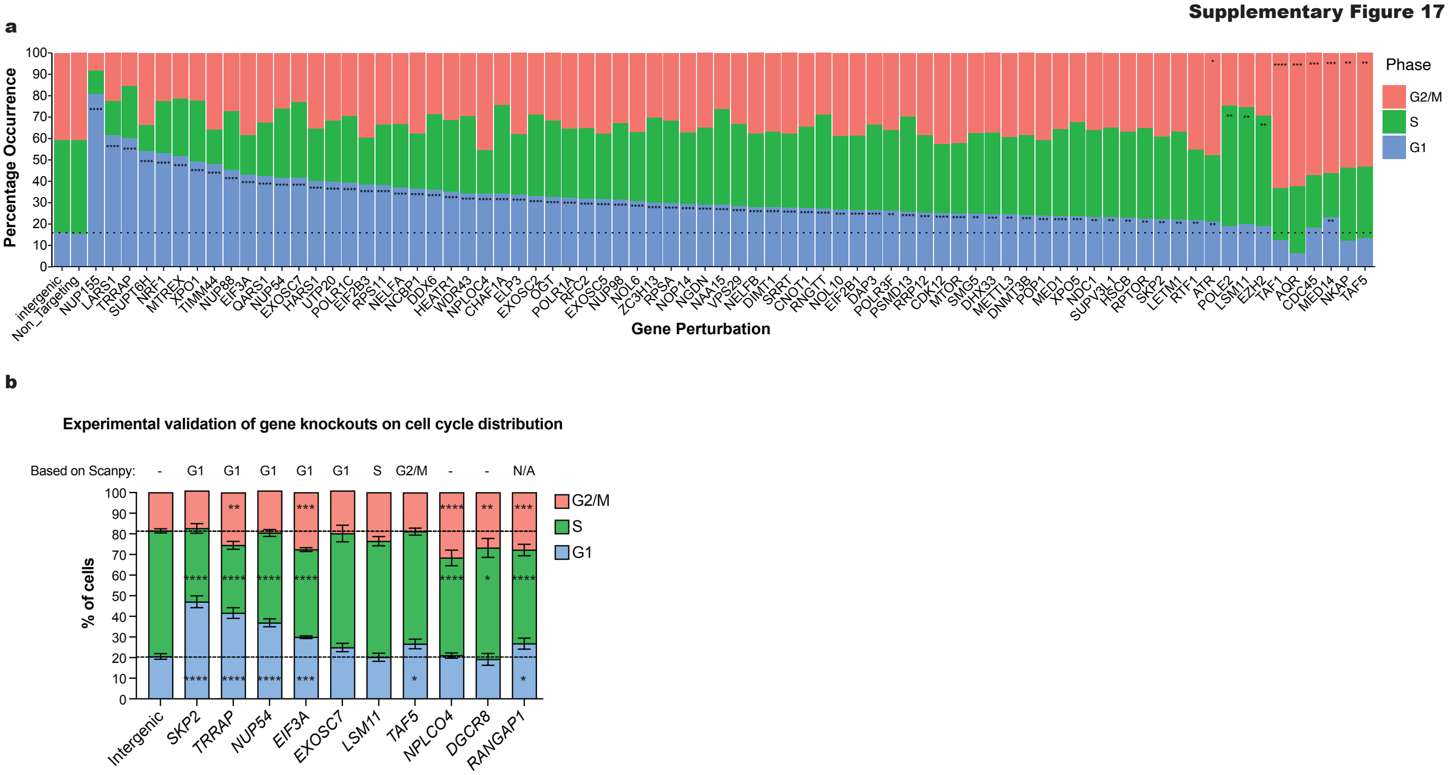

Supplementary Figure 17. scCHyMErA-Seq identifies genes influencing cell cycle distribution.
(a) Stacked bar plot showing genes identified by scCHyMErA-Seq as affecting cell cycle distribution. The cell cycle phase with a significantly increased fraction is indicated by stars ($p < 0.01$; Fisher's exact test, BH-corrected).
(b) Validation of scCHyMErA-Seq results by propidium iodide staining and flow cytometry. Stacked bar plots indicate the effect of gene knockouts on cell cycle distribution. Phases with significantly increased fractions are indicated (** $p < 0.01$, * $p < 0.05$; one-way ANOVA). Data represent mean ± SD from three biological replicates.

Supplementary Figure 18

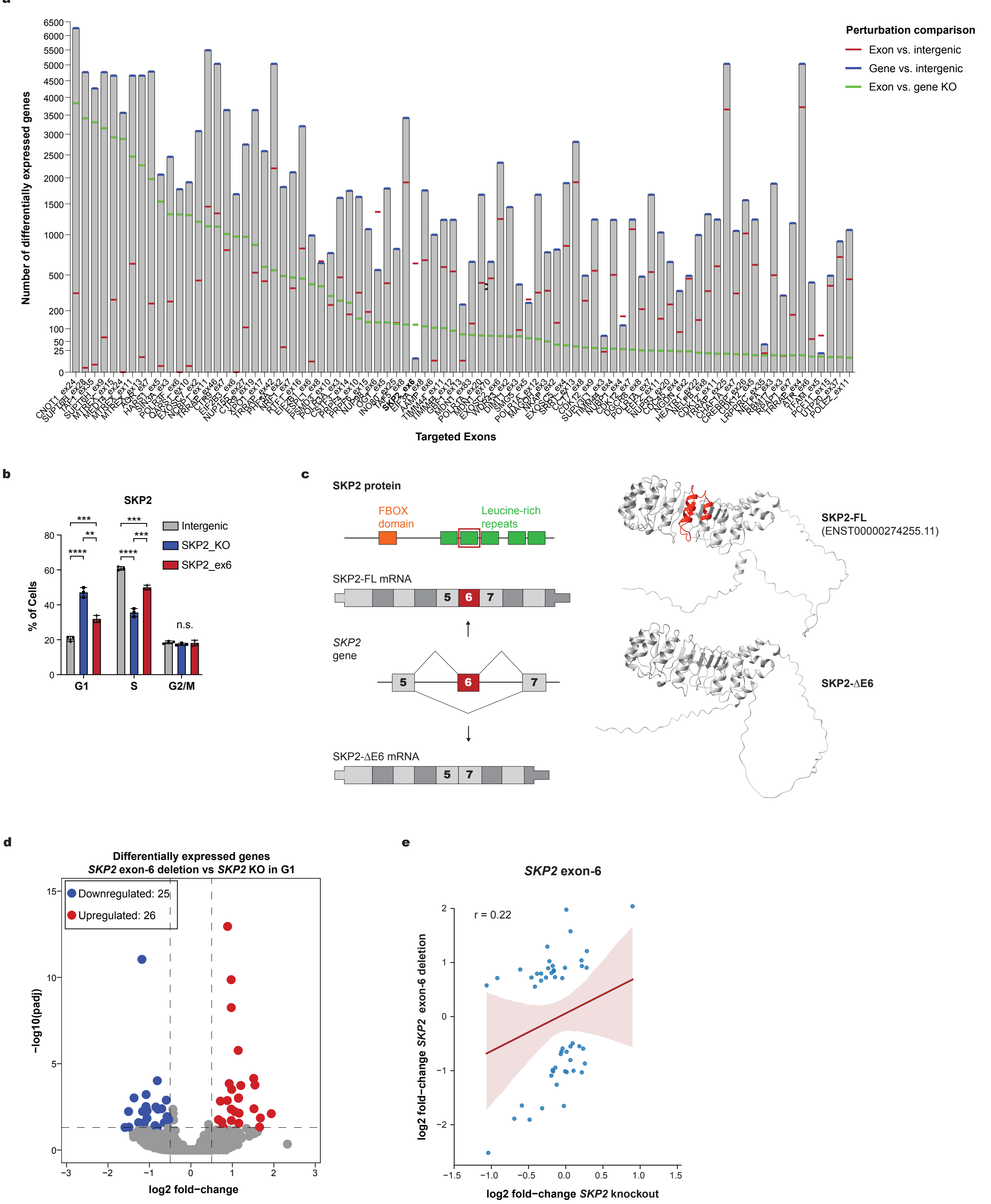

Supplementary Figure 18. Comparison of transcriptomic effects between gene knockout and exon deletion.
(a) Bar plot showing the number of differentially expressed genes identified by aggregate pseudobulk analysis across all perturbation types applied in the screen, comparing gene knockout vs exon deletion (green), gene knockout vs intergenic control (blue), and exon deletion vs intergenic control (red).
(b) Bar plot showing the distribution of cells across different cell cycle phases following transduction with intergenic control hgRNAs, or hgRNAs targeting SKP2 for knockout or exon-6 deletion. Bars represent mean ± SD. **** $p < 0.0001$, *** $p < 0.001$, ** $p < 0.01$; one-way ANOVA with Tukey's multiple comparisons test.
(c) Schematic of SKP2 protein domains and exon structure. The protein diagram (top) highlights the FBOX domain (orange), and leucine-rich repeats (green). The splicing schematic (middle) depicts the full-length (FL, top) and exon-6–deleted (ΔE6, bottom) mRNA isoforms. AlphaFold structural predictions[86, 87] for the SKP2 full-length (top right) and Δexon-6 (bottom right) isoforms are shown, with the exon-6-encoded region highlighted in red.
(d) Volcano plot of DESeq2 pseudobulk analysis comparing *SKP2* exon-6 deletion with *SKP2* knockout in G1-phase cells. Significantly upregulated genes are shown in red and downregulated genes in blue (adjusted $p < 0.05$; $|\log2 \text{ fold-change}| > 0.5$). p-values were computed using DESeq2's Wald test with Benjamini–Hochberg correction.
(e) Scatter plot comparing log2 fold-change values of genes between *SKP2* knockout vs intergenic controls (x-axis) and *SKP2* exon-6 deletion vs intergenic controls (y-axis) in G1-phase cells. Only genes differentially expressed between SKP2 knockout and exon-6 deletion are shown. Pearson correlation (r) is indicated.

Supplementary Figure 19

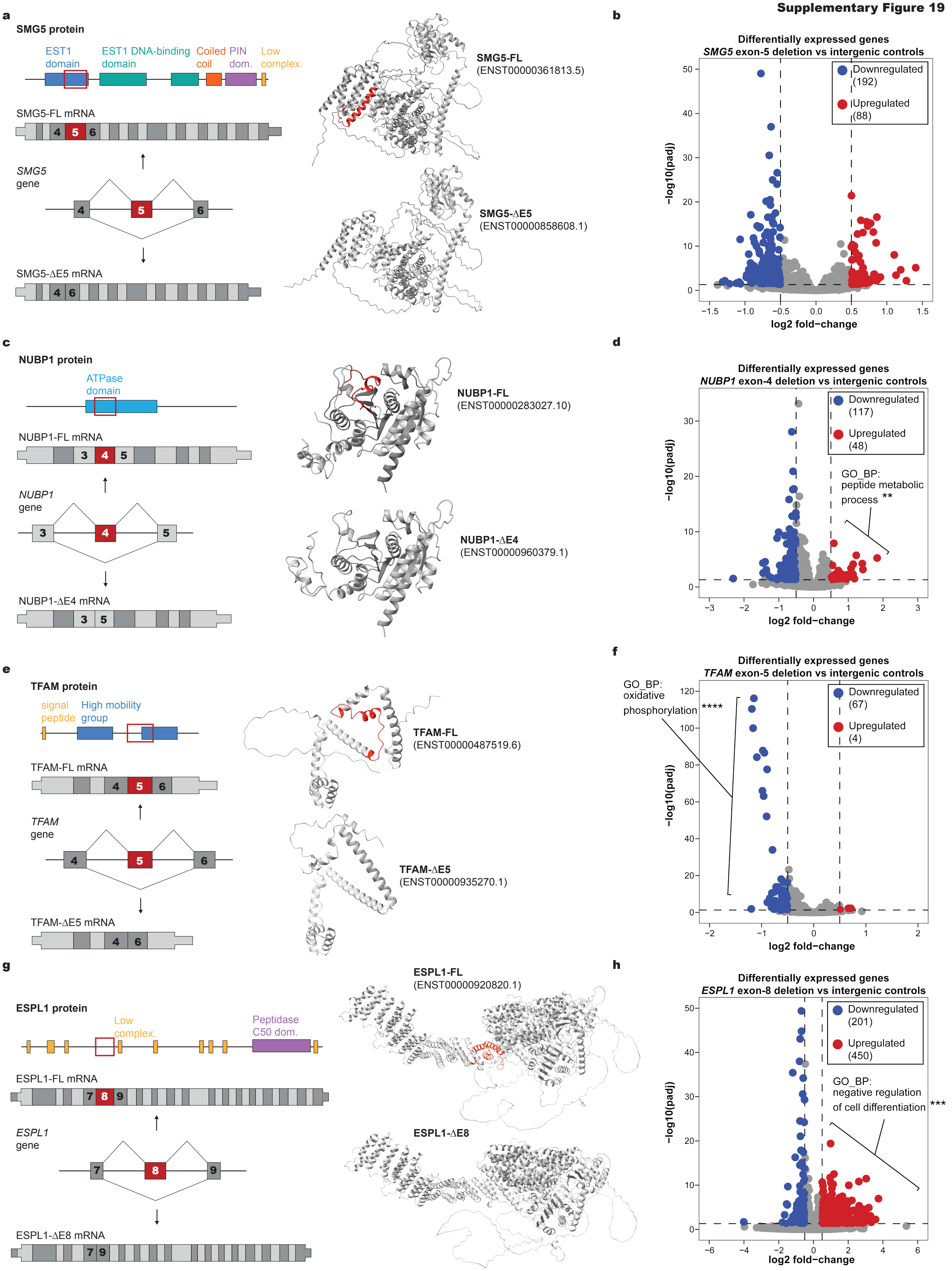

Supplementary Figure 19. Exon skipping vignettes with important predicted roles.
(a, c, e, g) Schematics of SMG5 (a), NUBP1 (c), TFAM (e), and ESPL1 (g) illustrating protein domain organization and exon structure. Protein diagrams (top) highlight annotated domains, while splicing schematics (middle) depict the full-length (FL, top) and exon-deleted (ΔE, bottom) mRNA isoforms. AlphaFold structural predictions[86, 87] for the full-length (top right) and Δexon (bottom right) isoforms are shown, with the region encoded by the skipped exon highlighted in red.
(b, d, f, h) Volcano plots showing DESeq2 pseudobulk differential expression analyses comparing cells carrying *SMG5* exon-5 (b), *NUBP1* exon-4 (d), *TFAM* exon-5 (f), or *ESPL1* exon-8 (h), deletions with cells transduced with non-targeting intergenic control hgRNAs. Each point represents a gene; significantly upregulated genes are shown in red and downregulated genes in blue (adjusted $p < 0.05$, $|\log 2$ fold change$| > 0.5$). p-values were computed using DESeq2's Wald test with Benjamini–Hochberg correction. For each comparison, the most significantly enriched Gene Ontology biological process (GO_BP) term (if any) is indicated separately for upregulated and downregulated genes, considering only terms that contain fewer than 300 genes. Fisher's exact one-tailed test with multiple-testing correction using g:Profiler's g:SCS method.

Supplementary Figure 20

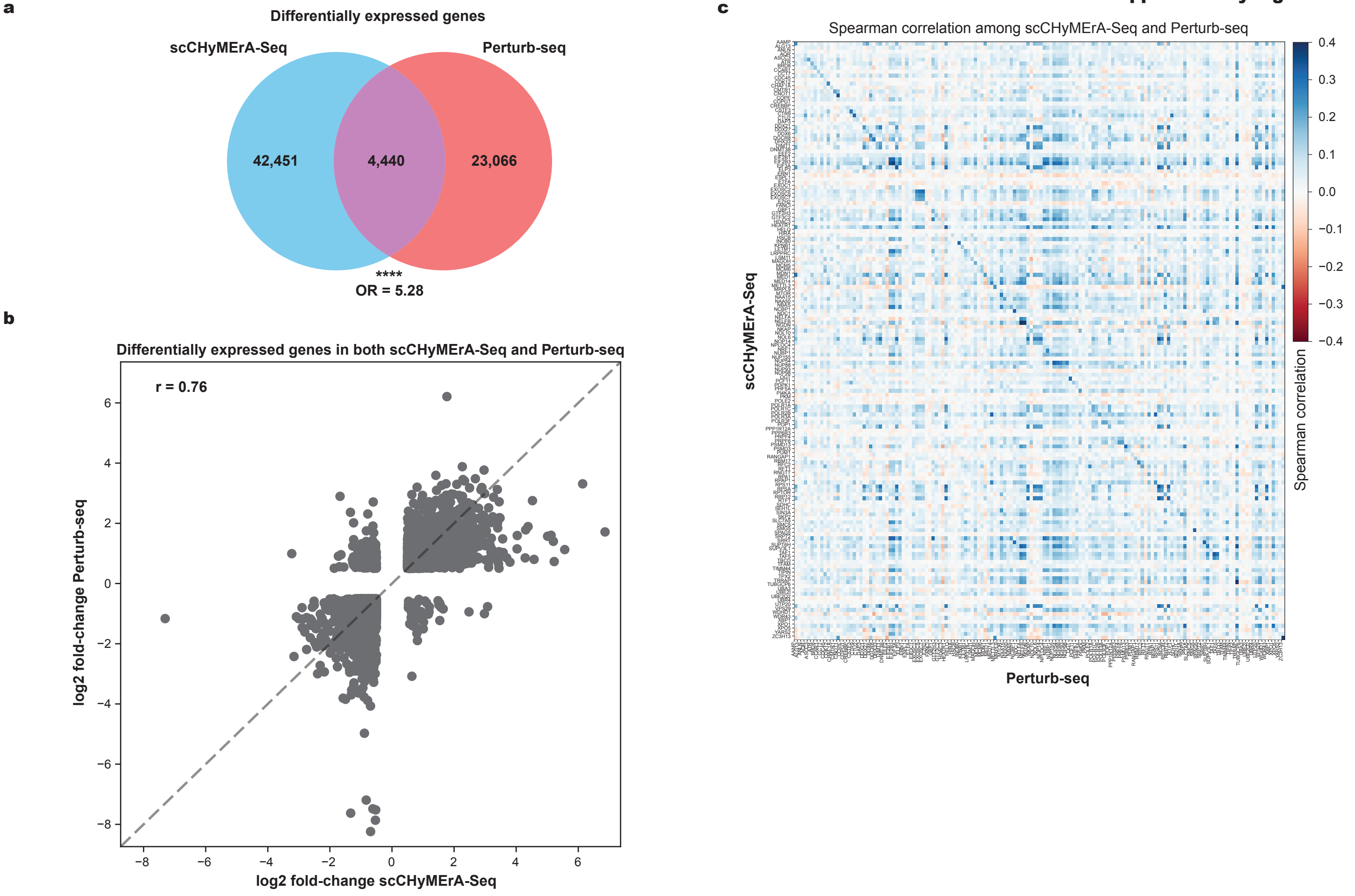

Supplementary Figure 20. Comparison of transcriptomic effects of gene inactivation between scCHyMErA-Seq and Perturb-seq.
(a) Venn diagram showing the overlap of differentially expressed genes identified following shared gene perturbations in scCHyMErA-Seq and Perturb-seq. Statistical significance was determined by two-sided Fisher's exact test (**** $p < 0.0001$), and the corresponding odds ratio (OR) is indicated.
(b) Scatter plot showing the Pearson correlation (r) of log2 fold-change values for differentially expressed genes identified by scCHyMErA-Seq and Perturb-seq. Each point represents a gene.
(c) Heatmap illustrating correlations between gene knockout profiles obtained with scCHyMErA-Seq and gene inactivation profiles from Perturb-seq. Color intensity represents Spearman correlation coefficients for differentially expressed genes across the corresponding hgRNA sequences.

a

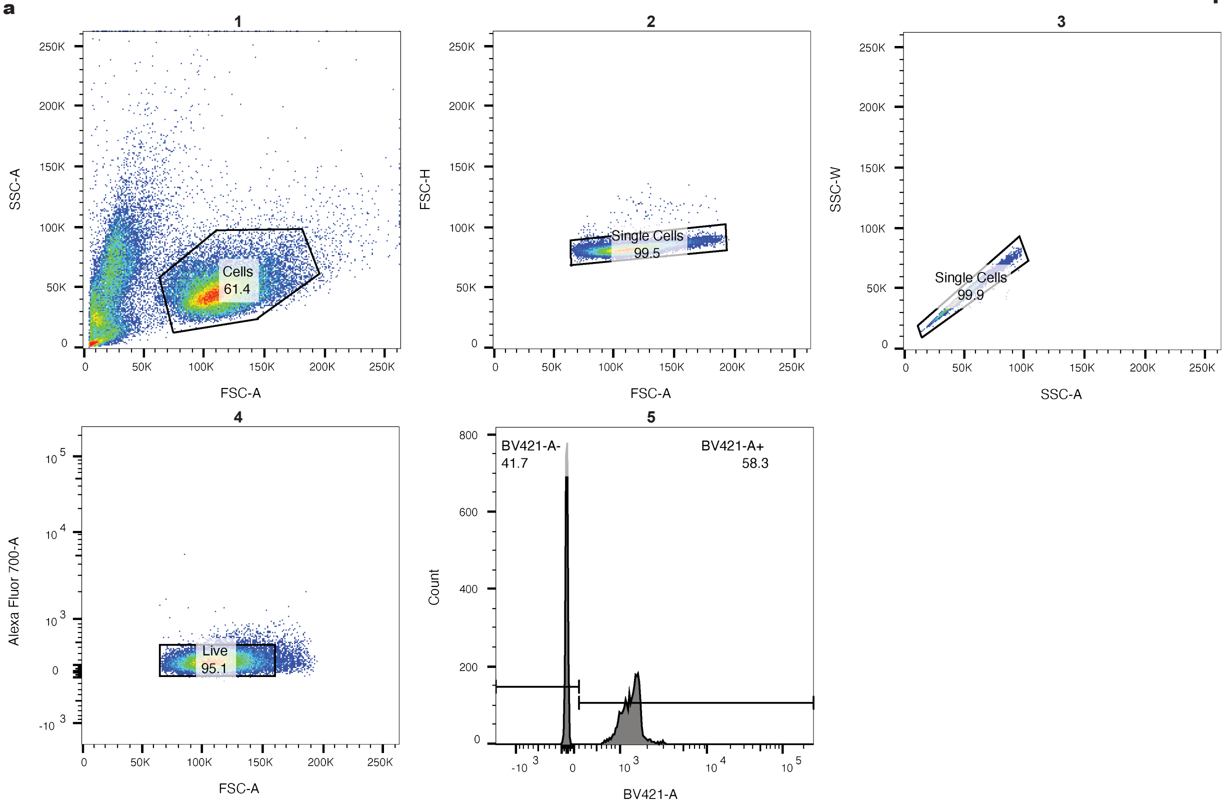

b

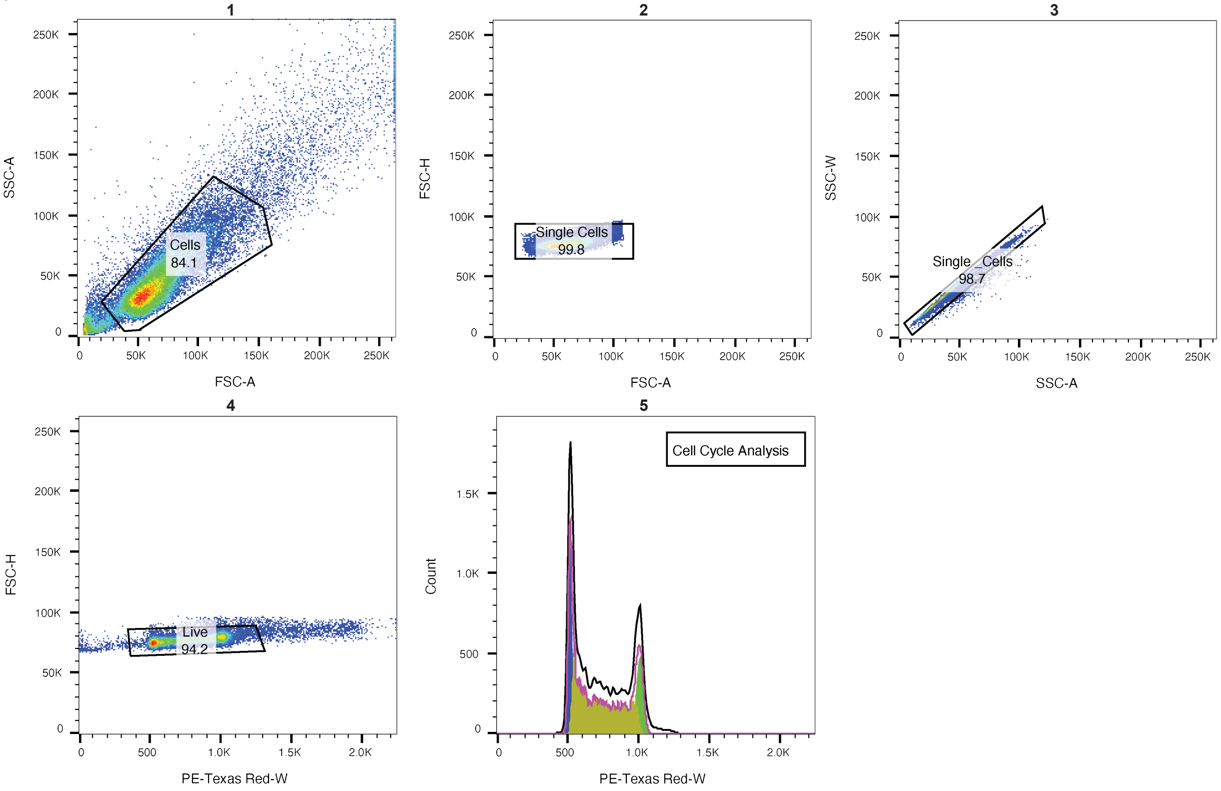

Supplementary Figure 21. Gating strategies for flow cytometry experiments.
(a-b) Sequential gating strategies used for flow cytometry analyses of (a) CD46 exon deletion, assessed using CD46–BV421 antibodies, and (b) cell cycle distribution, assessed by propidium iodide staining (PE–Texas Red). Numbers denote the order in which gates were applied during data analysis.
