# Supplementary material for: Single-cell exon deletion profiling reveals splicing events that shape gene expression and cell state dynamics
Source: Nat Commun. 2026 Feb 3;17:1218. doi: 10.1038/s41467-026-68774-w (PMC12868714; doi:10.1038/s41467-026-68774-w)
Supplement: Supplementary file 2 — Description of Additional Supplementary Files [file 41467_2026_68774_MOESM2_ESM.pdf]

## SUPPLEMENTARY DATA LEGENDS

**Supplementary Data 1. CRISPR gRNA capture efficiency using the 10x Genomics platform.** Summary of Cas9 and Cas12a gRNA capture efficiency across all constructs shown in Fig. 1. The total number of profiled cells, including those without detectable gRNA sequences, is also indicated.

**Supplementary Data 2. scCHyMErA-Seq exon deletion hgRNA library.**
Comprehensive sequence and annotation information for the scCHyMErA-Seq hgRNA screening library. Table includes targeted genes and exons, guide RNA sequences, predicted cut sites, on- and off-target scores, and corresponding library oligonucleotide sequences.

**Supplementary Data 3. Gene-level analysis of scCHyMErA-Seq data.**
**Sheet 1:** Summary of DESeq2 pseudobulk analyses comparing cells harboring individual gene knockouts to cells expressing non-targeting intergenic control hgRNAs. The total number of differentially expressed genes (DEGs), as well as the number of upregulated and downregulated genes for each perturbation, are provided.
**Sheet 2:** Complete list of DEGs identified in each gene-level perturbation, including log2 fold-change values and adjusted p-values.

**Supplementary Data 4. Exon deletion analyses of scCHyMErA-Seq data.**
**Sheet 1:** Summary of DESeq2 pseudobulk analyses comparing cells with specific exon deletions to cells expressing non-targeting intergenic control hgRNAs. The total number of differentially expressed genes (DEGs), along with counts of upregulated and downregulated genes for each perturbation, are provided.
**Sheet 2:** Complete list of DEGs identified in each exon deletion perturbation, including log2 fold-change values and adjusted p-values. p-values were computed using DESeq2's Wald test with Benjamini–Hochberg correction.

**Supplementary Data 5. NRF1 exon 7 deletion RNA-sequencing data.**
**Sheet 1:** DESeq2 analysis of HEK293 Flp-In cells depleted of endogenous NRF1 and rescued with either full-length (FL) or exon-7 deleted (ΔE7) NRF1 isoforms. Differentially expressed genes between siNRF1-NRF1-FL and siNRF1-NRF1-ΔE7 conditions are defined as those with $|\log2 \text{ fold-change}| > 0.5$ and adjusted $p\text{-value} < 0.05$.
**Sheet 2:** DESeq2 analysis of HEK293T cells transfected with three independent base editor constructs inducing NRF1 exon-7 skipping, or intergenic control gRNAs. Differentially expressed genes (adjusted $p\text{-value} < 0.05$ and $|\log2 \text{ fold-change}| > 0.5$) are defined as those with $|\log2 \text{ fold-change}| > 0.5$ and adjusted $p\text{-value} < 0.05$.

**Supplementary Data 6. Cell cycle analysis data.**
**Sheet 1:** Analysis of exon deletion scCHyMErA-Seq data showing the fraction of cells in different cell cycle phases following each exon deletion perturbation. Statistical analyses at the exon level were performed using one-sided Fisher's exact test (Benjamini–Hochberg-corrected) and the Mann-Whitney test (corrected for multiple hypothesis testing using the Benjamini–Hochberg method), with the three individual hgRNAs treated as replicates. Exons were considered to regulate the cell cycle only if significant by both approaches (Fisher's $p < 0.01$ and Mann-Whitney $p < 0.05$).
**Sheet 2:** Analysis of gene knockout scCHyMErA-Seq data showing the fraction of cells in different cell cycle phases following each gene knockout. Statistical analysis at the gene level was performed using one-sided Fisher's exact test (Benjamini–Hochberg-corrected). Genes with adjusted $p < 0.01$ were considered to regulate the cell cycle.

**Supplementary Data 7. Comparison of transcriptomic effects between gene knockout and exon deletion.**

**Sheet 1:** Summary of DESeq2 pseudobulk analyses comparing cells with specific exon deletions to cells expressing the corresponding gene knockout hgRNAs. The total number of differentially expressed genes (DEGs) is indicated, along with the number of DEGs following gene knockout or exon deletion compared to intergenic controls. Pearson correlation coefficients between gene- and exon-level phenotypes are also shown. For each targeted exon, the primary gene transcript ID and the longest exon skipping isoforms are provided, along with the number of amino acids encoded by each isoform.

**Sheet 2:** Complete list of DEGs identified for each exon deletion compared to the corresponding gene knockout, including log2 fold-change values and adjusted p-values. P-values were computed using DESeq2's Wald test with Benjamini–Hochberg correction.

**Supplementary Data 8. Oligonucleotides and Addgene plasmids used in this study.**

**Sheet 1:** Sequences of all oligonucleotides used, including hgRNA library constructs, primers, guide RNAs, and probes for northern blotting.

**Sheet 2:** List of newly cloned plasmids and libraries deposited to Addgene, including links to detailed descriptions and accession numbers.
